# Endonuclease G promotes hepatic mitochondrial respiration by selectively increasing mitochondrial tRNA^Thr^ production

**DOI:** 10.1073/pnas.2411298122

**Published:** 2025-01-03

**Authors:** Xihui Xu, Rozhin Penjweini, Lóránt Székvölgyi, Zsolt Karányi, Anne-Marie Heckel, Devikala Gurusamy, Dóra Varga, Shutong Yang, Alexandra L. Brown, Wenqi Cui, Jinsung Park, Dénes Nagy, Maren C. Podszun, Sarah Yang, Komudi Singh, Stephen P. Ashcroft, Jeonghan Kim, Myung K. Kim, Ivan Tarassov, Jun Zhu, Andrew Philp, Yaron Rotman, Jay R. Knutson, Nina Entelis, Jay H. Chung

**Affiliations:** ^a^Laboratory of Obesity and Aging Research, Cardiovascular Branch, National Heart Lung and Blood Institute, NIH, Bethesda, MD 20892; ^b^Laboratory of Advanced Microscopy and Biophotonics, Biochemistry and Biophysics Center, National Heart Lung and Blood Institute, NIH, Bethesda, MD 20892; ^c^Momentum Genome Architecture and Recombination Research Group, Department of Molecular and Nanopharmaceutics, Faculty of Pharmacy, University of Debrecen, Debrecen 4032, Hungary; ^d^Department of Internal Medicine, Faculty of Medicine, University of Debrecen, Debrecen 4032, Hungary; ^e^UMR 7156 Génétique Moléculaire, Génomique, Microbiologie, Strasbourg University-CNRS, Strasbourg 67000, France; ^f^Liver and Energy Metabolism Section, Liver Diseases Branch, National Institute of Diabetes and Digestive and Kidney Diseases, Bethesda, MD 20892; ^g^DNA Sequencing and Genomics Core Facility, National Heart Lung and Blood Institute, NIH, Bethesda, MD 20892; ^h^Bioinformatics Core Facility, National Heart Lung and Blood Institute, NIH, Bethesda, MD 20892; ^i^School of Sport, Exercise and Rehabilitation Sciences, University of Birmingham, Birmingham B152TT, United Kingdom; ^j^Department of Biochemistry, College of Medicine, The Catholic University of Korea, Seoul 06591, South Korea; ^k^Department of Medical Sciences, Graduate School of The Catholic University of Korea, Seoul 06591, South Korea; ^l^Centre for Healthy Ageing, Centenary Institute, Royal Prince Alfred Hospital, Sydney, NSW 2050, Australia; ^m^School of Sport, Exercise and Rehabilitation Sciences, University of Technology Sydney, Sydney, NSW 2007, Australia

**Keywords:** endonuclease G, mitochondria, MASLD, tRNA

## Abstract

Mitochondrial endonuclease G (EndoG) contributes to chromosomal degradation when it is released from mitochondria during apoptosis. It is presumed to also have a mitochondrial function because EndoG deficiency causes mitochondrial dysfunction. However, the mechanism by which EndoG regulates mitochondrial function is not known. Fat accumulation in metabolic dysfunction–associated steatotic liver disease (MASLD), which is more common in men, is caused in part by mitochondrial dysfunction. EndoG expression is reduced in MASLD liver, and EndoG deficiency causes MASLD in an obesity-independent manner but only in males. EndoG promotes mitochondrial respiration by resolving mitochondrial tRNA/DNA hybrids formed during mtDNA transcription by recruiting RNA helicase DHX30 to unwind them. EndoG also cleaves off the 3′-end of the H-strand transcript that can prevent mt-tRNA^Thr^ precursor cloverleaf-folding, and processing, which increases mt-tRNA^Thr^ production and mitochondrial translation. Using fluorescent lifetime imaging microscopy technology to visualize oxygen consumption at the individual mitochondrion level, we found that EndoG deficiency leads to the selective loss of a mitochondrial subpopulation with high-oxygen consumption. This defect was reversed with mt-tRNA^Thr^ supplementation. Thus, EndoG promotes mitochondrial respiration by selectively regulating the production of mt-tRNA^Thr^ in male mice.

## Significance

Metabolic dysfunction-associated steatotic liver disease (MASLD) affects 25 to 35% of the adult population in developed nations, making it a significant public health crisis. In this paper, we provide evidence that decreased levels of mitochondrial endonuclease G contribute to obesity-independent MASLD in male mice. We found that endonuclease G (EndoG) deficiency leads to liver-specific fat accumulation in mice, independent of body weight. EndoG protects against MASLD by promoting mitochondrial threonine transfer RNA (tRNA^Thr^) processing by cleaving the 3′ end of the heavy-strand mitochondrial transcript. This is an example of a mitochondrial nuclease regulating oxidative phosphorylation without affecting mitochondrial DNA (mtDNA) content or stability. Moreover, our study suggests that increasing hepatic EndoG activity or mt-tRNA^Thr^ levels may be a therapeutic strategy to ameliorate MASLD.

Nuclear-encoded endonuclease G (EndoG) is mostly localized in mitochondria (1, 2) and cleaves C/G-rich RNA, DNA, and RNA/DNA hybrids (1, 3). In addition, it has high cleavage activity on aberrant nucleic structures such as G-quadruplex (4), kinked DNA and 5-hydroxymethylcytosine, an oxidized variant of cytosine (5), indicating that it is a semi–sequence/structure-specific nuclease. It was first discovered as a nuclease that translocates to the nucleus and degrades chromosomal DNA during apoptosis (6). Its cleavage of RNA in the regulatory D-loop region of mitochondrial genome has been proposed to generate RNA primers for mtDNA replication (7). It has also been reported that EndoG can be released from mitochondria during starvation to inhibit mTOR and promote autophagy (8). Recently, EndoG deficiency has been reported to decrease body weight and protect against metabolic dysfunction–associated steatotic liver disease (MASLD) in female mice, but not in male mice (9). In *Caenorhabditis elegans*, EndoG degrades the paternal mtDNA after fertilization (10)‚ but its intramitochondrial function in vertebrates may be more complicated as invertebrates and vertebrates have different EndoG nuclease profiles: In invertebrates, the EndoG ortholog has both endonuclease and exonuclease activities, whereas in vertebrates, these activities are divided between two separated, but related, nucleases: EndoG and Exonuclease G (11). EndoG KO mice also develop mitochondrial abnormalities in the heart: hypertrophy, mitochondrial dysfunction, and oxidative stress (12), but the intramitochondrial function of EndoG is not known.

Approximately 25 to 35% of population in developed nations develop MASLD, which ranges on a spectrum from simple hepatic steatosis (HS), characterized by hepatic triglyceride accumulation that can progress to nonalcoholic steatohepatitis, cirrhosis, and, ultimately, hepatocellular carcinoma (13). Obesity is a leading risk factor for HS, but it is not rare even in lean individuals (14, 15), indicating that obesity-independent causes of HS may exist. Interestingly, MASLD is more common in men compared to women; the reason for the sexual dimorphism is not known. Since mitochondrial dysfunction (16) and oxidative stress (17) are important features of the MASLD, we investigated the potential role of EndoG in mitochondrial function.

Here, we show that EndoG deficiency causes MASLD in male mice but not female mice. EndoG promotes mitochondrial respiration by resolving mitochondrial tRNA/DNA hybrids (R-loops) (18) formed during mtDNA transcription through recruitment of RNA helicase DHX30 (19) to unwind mitochondrial R-loops. EndoG also cleaves off the 3′-end of the mtDNA H-strand transcript which can block mt-tRNA^Thr^ precursor cloverleaf-folding and its further processing, and therefore, mature mt-tRNA^Thr^ production. Consequently, decreased EndoG reduces mt-tRNA^Thr^ steady-state level, reducing intramitochondrial protein synthesis and mitochondrial respiration. Analyses of the oxygen concentration at the individual mitochondrion level by fluorescent life-time microscopy (FLIM) (20, 21) show that EndoG KO leads to the loss of the high-oxygen consumption mitochondrial subpopulation. Supplementation of mt-tRNA^Thr^ restores the high-oxygen consumption mitochondrial subpopulation which is lacking in EndoG KO cells. Therefore, we conclude that endonuclease G promotes hepatic mitochondrial respiration by selectively increasing mitochondrial tRNA^Thr^ production.

## Results

### EndoG Deficiency Leads to Hepatic Steatosis.

**Fig. 1.**
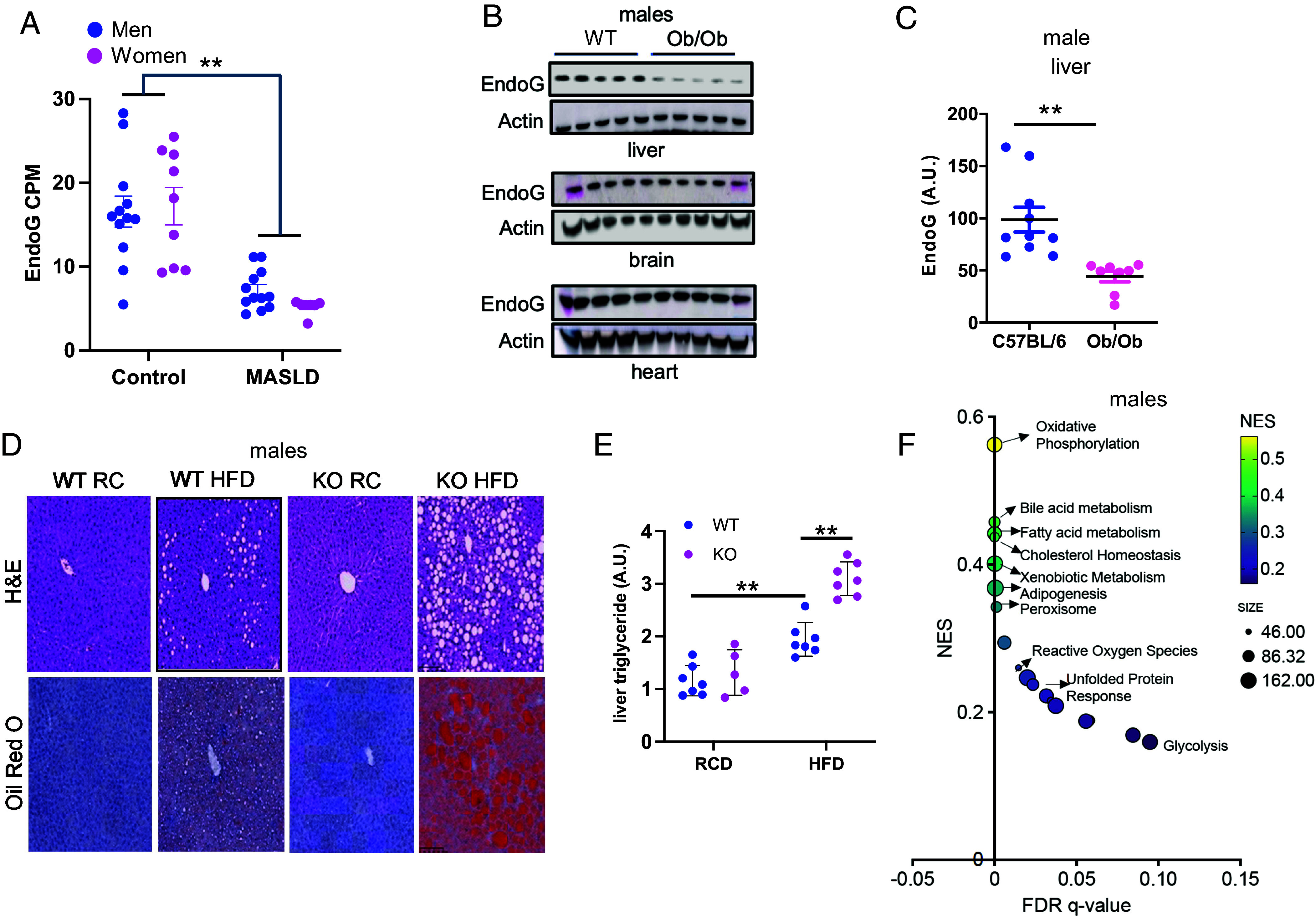
EndoG deficiency leads to HS. (*A*) *EndoG* mRNA levels in liver tissues obtained by needle biopsy from healthy controls and the MASLD individuals (n = 19). (*B*) EndoG protein expression in tissues of the MASLD-prone male *ob/ob* mice and WT (C57BL6/J) mice. (*C*) Quantification of the hepatic EndoG levels in WT and *ob/ob* mice (n = 10). (*D*) Representative images of H&E (*Upper*) and Oil Red O (*Lower*) staining of the liver sections of male WT and EndoG KO mice on RC or HFD for 3 mo after weaning. (*E*) Hepatic triglyceride levels of male EndoG WT and KO mice on RC or HFD (n = 5-7). (*F*) RNA-seq of WT and EndoG KO liver. GSEA using Hallmark gene sets demonstrates positive enrichment of genes related to the specified pathways. The normalized enrichment score (NES) is plotted against the false discovery rate (FDR) q-value, with the size of the circles indicating the number of genes enriched in the gene analysis sets. Results are shown as the mean ± SEM. **P < 0.01.

RNA sequencing (RNA-seq) analysis was performed on liver tissue samples from subjects with MASLD and controls (22). *ENDOG* was one of the mitochondria-localized genes whose expression decreased in MASLD (Fig. 1*A*). In order to determine whether decreased EndoG in MASLD subjects is causational, we studied mouse models of MASLD. One such model is *ob/ob* mice, which lack the adipokine leptin and, as a result, develop hyperphagia, severe obesity, and MASLD (23). We compared EndoG levels in the liver, heart, and brain of male *ob/ob* with those of lean wild-type (WT) mice (Fig. 1 *B* and *C*). EndoG levels were reduced in the liver of male ob/ob (*P* < 0.01) and female (*P* = 0.51) *ob/ob* mice (*SI Appendix*, Fig. S1).

To evaluate whether the reduction in hepatic EndoG levels was simply a consequence of HS or could be a cause of HS, we visualized hepatic lipids in young (3 mo) and middle age (10 mo) male EndoG WT and KO mice. EndoG KO and WT mice had similar hepatic lipid content at 3 mo, but EndoG KO had significantly more hepatic lipid content at 10 mo (*SI Appendix*, Fig. S2*A*). Consistent with this, EndoG KO had higher serum triglyceride and liver damage at 10 mo (*SI Appendix*, Fig. S2*B*). Young male EndoG KO mice fed high fat diet (HFD) for 3 mo also developed increased hepatic triglyceride levels compared to their EndoG WT counterparts (Fig. 1 *D* and *E*). However, in contrast to male EndoG KO mice, female EndoG KO mice demonstrated lower body weight and reduced hepatic triglyceride levels when subjected to a high-fat diet (HFD) (*SI Appendix*, Fig. S2*C*) Therefore, we focused on understanding the mechanism of MASLD in male EndoG KO mice. Unless stated otherwise (*SI Appendix*, Figs. S1 and S2 *C* and D), hereafter, all figures and texts refer to male mice. We began by performing RNA-seq and Gene set enrichment analysis (GSEA) on livers from male mice fed HFD. The analysis revealed that the pathway most affected in EndoG KO liver is oxidative phosphorylation, indicating that male EndoG mice have defective mitochondria in the liver (Fig. 1*F* and *SI Appendix*, Fig. S2*D*).

Despite the difference in hepatic lipids, male EndoG WT and KO mice had similar accumulation of fat in other tissues, including the heart (*SI Appendix*, Fig. S3). They also had similar body weight (*SI Appendix*, Fig. S4*A*) and food intake (*SI Appendix*, Fig. S4 *B* and C) on both the regular chow (RC) and HFD. Even at 10 mo, they had similar body weight (*SI Appendix*, Fig. S4*D*), total body fat content (*SI Appendix*, Fig. S4 *E* and F), and lean mass (*SI Appendix*, Fig. S4 *G* and H). The levels of hepatic *ENDOG* mRNA also did not correlate with BMI (*SI Appendix*, Fig. S4*I*) in humans. Taken together, these findings indicate that EndoG deficiency causes HS independent of food intake and body weight in male mice.

### EndoG Regulates Mitochondrial Respiration.

**Fig. 2.**
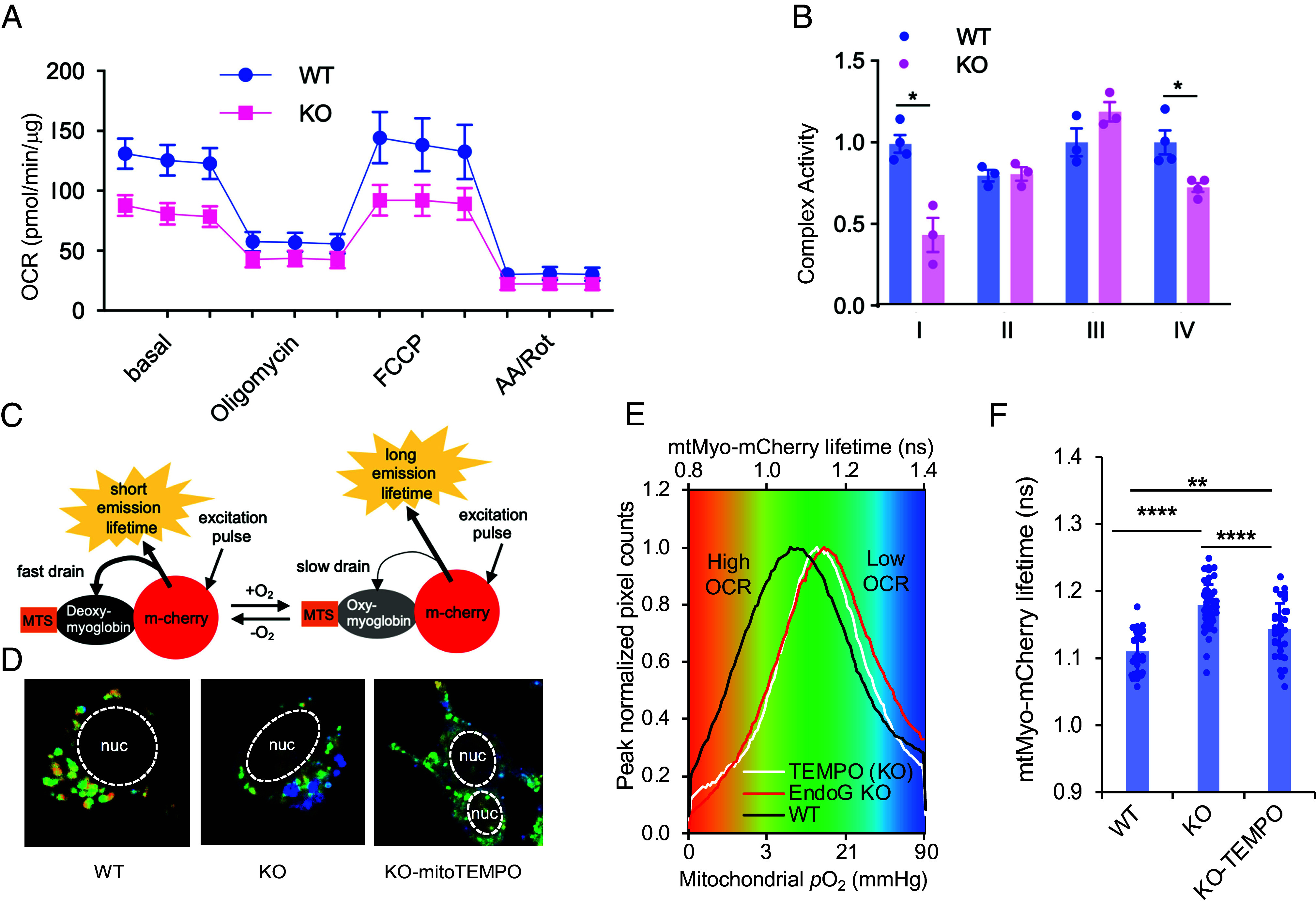
EndoG regulates mitochondrial respiration. (*A*) OCR of EndoG WT and KO MEFs as measured by the Seahorse mitochondrial stress test. (*B*) The ETC complex activity of mitochondrial complexes I-IV in WT and KO MEFs. (*C*) Schematic diagram showing the construct of the mitochondrial O_2_ probe we designed for FLIM and how FLIM works to detect mitochondrial [O_2_]. MTS, mitochondrial targeting sequence. Deoxy-myoglobin, deoxygenated myoglobin; Oxy-myoglobin, oxygenated myoglobin. (*D*) Representative confocal images showing pseudocolor mapping of mtMyo-mCherry fluorescence lifetime in EndoG WT and KO MEFs and KO MEFs treated with mito-TEMPO. (*E*) Fluorescence lifetime distribution histogram of mtMyo-mCherry in mitochondria; red indicates a shorter lifetime with lower [O_2_] (high OCR) and blue indicates a longer lifetime with higher [O_2_] (low OCR). (*F*) Quantification of the lifetimes shown in (*E*) (n ≥ 20). Results are shown as the mean ± SEM. *P < 0.05; **P < 0.01; ****P < 0.0001.

To confirm the presence of a defect in fatty acid oxidation in EndoG KO, we challenged mouse embryo fibroblasts (MEFs) with long-chain fatty acids that have been conjugated to bovine serum albumin (BSA). EndoG KO MEFs accumulated more lipid after oleic acid treatment as measured by confocal microscopy and flow cytometry (*SI Appendix*, Fig. S5 *A*–C). They also had higher cell death rate after 24 h treatment with palmitic acid (*SI Appendix*, Fig. S5*D*). EndoG KO cells had lower average oxygen consumption rate (OCR) by the Seahorse assay (Fig. 2*A*) and lower oxidative phosphorylation (OXPHOS) enzymatic activity in complexes I and IV but not II and III (Fig. 2*B*). These findings are consistent with the notion that EndoG deficiency leads to a defect in fatty acid metabolism which requires OXPHOS.

Each cell contains a network of hundreds of mitochondria, with each one having different OXPHOS activity as measured by OCR (20). Given the heterogeneity of the mitochondrial OCR, the distribution profile of OCR can provide deeper insights into mitochondrial function that cannot be provided by the Seahorse XF technology or mitochondrial complex activity measurement. For this purpose, we modified a noninvasive and highly sensitive two-photon FLIM to visualize the OCR at a single mitochondrion level (20, 21). The [O_2_] probe is a Förster Resonance Energy Transfer (FRET)-based probe consisting of myoglobin fused to mCherry red fluorescent protein (Myo-mCherry). A mitochondrial targeting sequence was added to Myo-mCherry to localize it to mitochondria. The overlap between the absorption spectrum of myoglobin in 590 to 650 nm and the emission of mCherry determines the energy that mCherry transfers to myoglobin by FRET and, thus, the lifetime of mCherry fluorescence. The emission spectrum of mCherry overlaps more with the absorption spectrum of deoxygenated (deoxy-) myoglobin compared to its oxygenated (oxy-) form (Fig. 2*C*). The pixel-by-pixel pO_2_, which correlates inversely to OCR, was then visualized by pseudocoloring (orange for low-pO_2_/high-OCR to blue for high-pO_2_/low-OCR) (Fig. 2*D*). EndoG KO MEFs had significantly reduced high-OCR mitochondrial subpopulation and increased low-OCR subpopulation than WT MEFs (Fig. 2 *E* and *F*). As EndoG KO MEFs and liver produced more mitochondrial reactive oxygen species (mROS) than WT liver (*SI Appendix*, Fig. S6*A*), mROS may repress OCR in EndoG KO MEFs. However, treatment of EndoG KO MEFs with mitochondrial antioxidant mito-TEMPO reduced the very low-OCR subpopulation but did not increase the overall OCR except for a small, very high-OCR subpopulation (Fig. 2 *E* and *F*). Our data show decreased high-OCR mitochondrial subpopulation in EndoG KO MEFs that is not caused by increased mROS.

### EndoG Increases Mitochondrial Translation by Promoting mt-tRNA^Thr^ Production.

EndoG has been implicated in mtDNA repair (5) and/or replication (7, 24). These reports, along with the observed increase in mROS in EndoG KO liver (*SI Appendix*, Fig. S6*A*), suggest that the mitochondrial dysfunction in EndoG KO cells might be a consequence of increased mtDNA damage. Therefore, we sequenced liver mtDNA isolated from 12 mo mice to maximize our chance of detecting mtDNA mutations. Surprisingly, there was no significant difference in either the total mtDNA copy number (*SI Appendix*, Fig. S6*B*) or the frequency of heteroplasmic mutations, large insertions and deletions (InDels), or intrachromosomal translocations between them (*SI Appendix*, Fig. S6 *C*–G). Therefore, EndoG does not appear to be critical for either mtDNA replication or maintaining the integrity of the mitochondrial genome, suggesting that the OXPHOS dysfunction in EndoG KO is not caused by increased mtDNA damage.

**Fig. 3.**
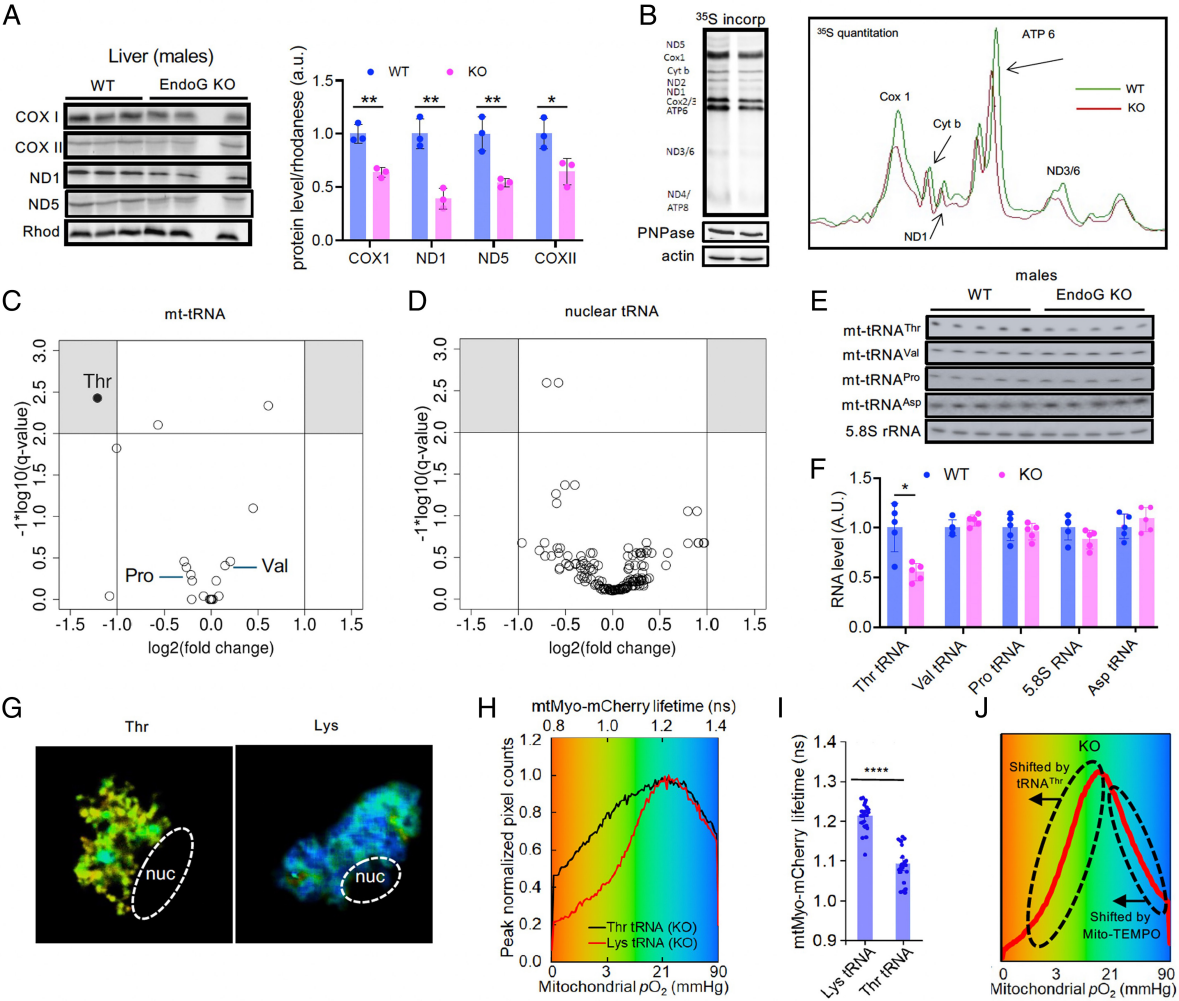
EndoG increases translation of the mitochondrial genome by promoting mt-tRNA^Thr^ production. (*A*) (*Left*) Proteins that are translated in mitochondria are shown along with Rhodanese (Rhod), a protein that is translated in the cytoplasm prior to mitochondrial translocation (control). (*Right*) Quantification of the protein levels in EndoG WT and KO livers are shown on the *Right* (n = 3). (*B*) Mitochondrial translation is visualized by ^35^S-methionine incorporation into mitochondria of EndoG WT and KO MEFs. Polynucleotide phosphorylase (PNPase), which is in mitochondria but translated in the cytoplasm, and actin are shown at the *Bottom* as controls (*Left*). A representative image of the densitometric analysis of three independent experiments is shown on the *Right*. (*C*) Volcano plot of tRNA-Seq analysis of mt-tRNAs in liver tissues from 2.5 mo EndoG WT and KO mice showing that mt-tRNA^Thr^ is decreased in EndoG KO mice compared to EndoG WT mice (n = 3). The gray boxes in upper corners indicate statistically significant changes. There are six tRNAs that are all overlapping at zerofold change. (*D*) Volcano plot of tRNA-Seq analysis of nuclear-encoded tRNAs (n = 3). (*E*) Northern blots showing mt-tRNA^Thr^, mt-tRNA^Val^, mt-tRNA^Pro^, mt-tRNA^Asp^ as well as cytoplasmic 5.8S rRNA in EndoG WT and KO liver. (*F*) Quantification of panel (*E*) (n = 5). (*G*) Pseudocolored FLIM images of EndoG KO MEFs after transfection with either mt-tRNA^Thr^ or mt-tRNA^Lys^ (control). (*H*) Histogram of FLIM shown in (*G*). (*I*) Quantification of the mtMyo-mCherry lifetime shown in (*H*) (n ≥ 20). (*J*) Summary of how different mitochondrial subpopulations in EndoG KO cells is affected differently by mt-tRNA^Thr^ supplementation and mito-TEMPO. Results are shown as the mean ± SEM. *P < 0.05; **P <0.01; ****P <0.0001.

We then investigated whether EndoG deficiency affected the levels of the 13 mtDNA encoded proteins, all of which are subunits of the OXPHOS system. Our attempts to quantify them with mass spectrometry yielded very inconsistent results due to their high lipophilicity. Most commercially available antibodies were found to lack specificity, but we were able to find highly specific antibodies for four of the 13 polypeptides: COX I, COX II, ND1, and ND5. We performed immunoblotting of liver lysates from 2 mo male EndoG WT and KO mice, before the development of HS, and found that the levels of all four polypeptides were lower in EndoG KO mice (Fig. 3*A*) while those of the nucleus-encoded mitochondrial protein Rhodanese were similar.

Due to its RNase and RNase H activity, we investigated the effect of EndoG on mitochondrial and nuclear transcription as well as on RNA stability with RNA-seq in the livers of 2 mo mice. Except for the absence of EndoG mRNA, the KO liver had a very similar transcriptome to that of the WT liver (*SI Appendix*, Fig. S7*A*). Furthermore, reverse-transcription qPCR (rt-qPCR) of the 13 mitochondrial mRNAs also showed no difference between EndoG WT and KO livers (*SI Appendix*, Fig. S7*B*). This prompted us to quantify mitochondrial protein synthesis by measuring mitochondrial pulse–chase incorporation of ^35^S-methionine in EndoG WT and KO MEFs. Compared to EndoG WT MEFs, KO MEFs had lower ^35^S-methionine incorporation into mitochondria-encoded proteins (Fig. 3*B*), indicating that EndoG KO mitochondria have a translational defect.

Mitochondrial-RNAs are transcribed as long polycistronic transcripts from the heavy (H) and light (L) strands of mtDNA. In addition to 13 mRNAs and two rRNAs, the mitochondrial genome encodes 22 tRNAs which are mostly positioned between protein-coding genes. They serve as punctuation marks between mRNAs (25) in the polycistronic transcripts. We hypothesized that EndoG deficiency causes mt-tRNA defects, which are known to cause mitochondrial dysfunction (26, 27). Unlike mRNAs and rRNAs, tRNAs cannot be quantified accurately with RNA-seq or rt-qPCR due to heavy posttranscriptional modifications, including methylation. We performed tRNA-seq after demethylation of RNA (28, 29) and found that EndoG KO liver had reduced mt-tRNA^Thr^ levels (Fig. 3*C*) even though there was no difference in nuclear-encoded tRNAs (Fig. 3*D*). Northern hybridization using mt-tRNA-specific probes confirmed that mt-tRNA^Thr^ levels were reduced in EndoG KO livers but not that of mt-tRNA^Val^, mt-tRNA^Asp^, or mt-tRNA^Pro^ (Fig. 3 *E* and *F*).

If mt-tRNA^Thr^ is rate-limiting for OXPHOS in EndoG KO MEFs, then delivery of additional tRNA^Thr^ into their mitochondria should normalize the OCR profile. The delivery of cytosolic tRNA into mitochondria requires certain structural features in the tRNA, which limits the repertoire of tRNAs that can be delivered (30). However, we previously demonstrated that human mitochondrial tRNA^Lys^ introduced in cytosol is translocated into mitochondria and produced curative effect (31, 32). Importantly, mt-tRNA^Thr^ was also imported into mitochondria as visualized by northern hybridization of mitoplast RNA after cellular transfection (*SI Appendix*, Fig. S8*A*). FLIM analysis after transiently transfecting EndoG KO MEFs with either mt-tRNA^Thr^ or mt-tRNA^Lys^ (control) indicated that mt-tRNA^Thr^ reversed most of OCR defect in EndoG KO MEFs compared to mt-tRNA^Lys^ (Fig. 3 *G*–*I*). Interestingly, the low-OCR subpopulation, which was reduced by mito-TEMPO (Fig. 2 *D*–*F*), was not reduced by mt-tRNA^Thr^ transfection. Thus, the low-OCR and high-OCR mitochondrial subpopulations in EndoG KO have different defects (summarized in Fig. 3*J*). Since *ob/ob* livers have reduced EndoG (Fig. 1 *B* and *C*), we performed northern hybridization with mt-tRNA probes and found that mt-tRNA^Thr^ was selectively reduced in *ob/ob* livers (*SI Appendix*, Fig. S8*B*). Taken together, these findings indicate that the OXPHOS defect in EndoG KO mitochondria is caused by mt-tRNA^Thr^ insufficiency.

### EndoG Promotes R-Loop Resolution by Recruiting DHX30 Helicase.

**Fig. 4.**
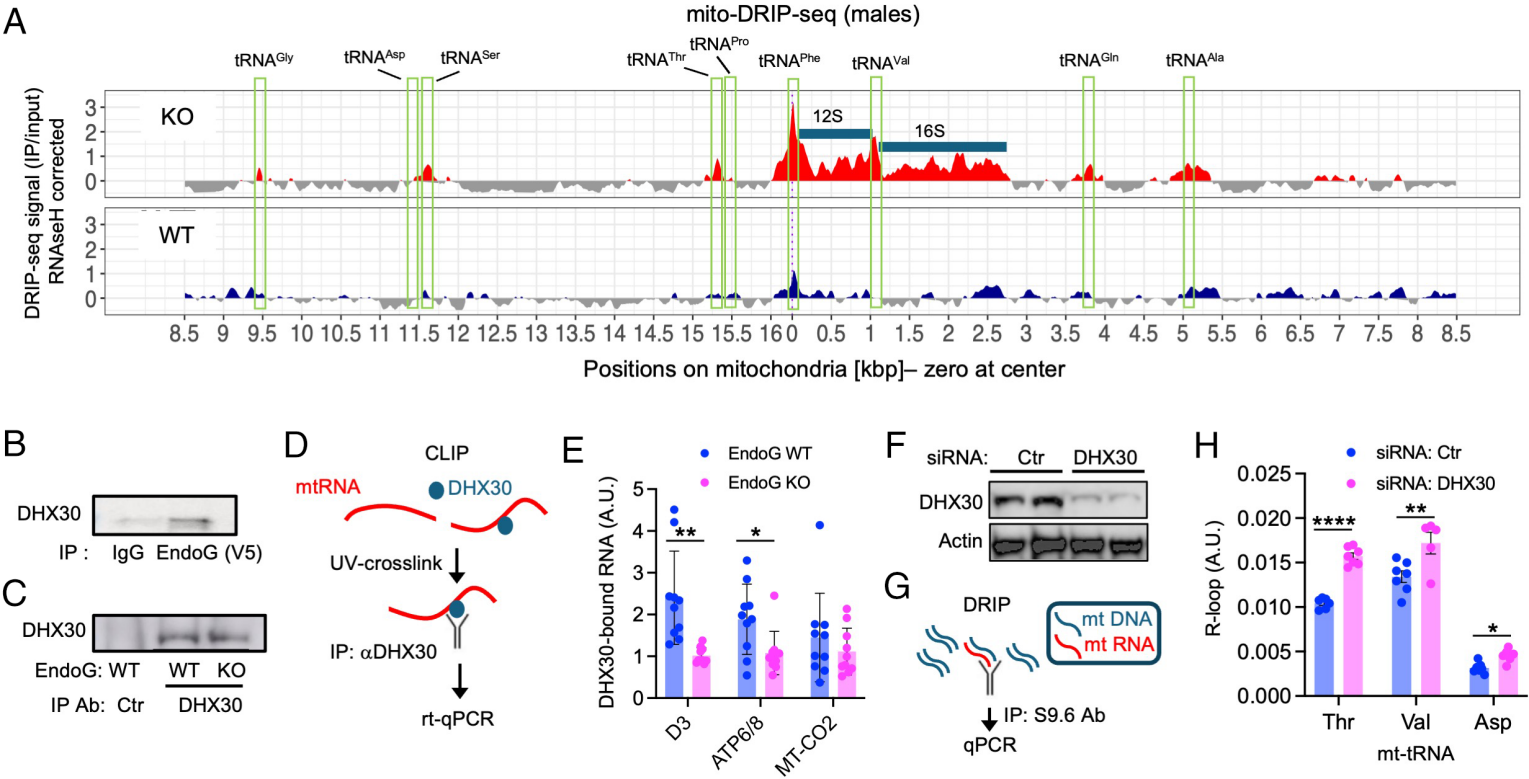
EndoG promotes R-loop resolution by recruiting DHX30 helicase. (*A*) Mitochondrial RNA-DNA hybrid immunoprecipitation sequencing (mito-DRIP-seq) data show increased R-loops mapping to a number of mt-tRNAs (green box) and both mt-rRNAs (blue bar) in EndoG KO liver compared to EndoG WT liver. The Y axis shows the enrichment of input-normalized DRIP signal compared to RNaseH1-treated control samples (35). (*B*) Coimmunoprecipitation between EndoG and DHX30. (*C*) Immunoprecipitation of DHX30 after UV-crosslinking in EndoG WT and KO MEFs prior to CLIP assay. (*D*) Schematic diagram of CLIP assay. (*E*) RT-qPCR quantification of mt-RNA bound to DHX30 in EndoG WT and KO MEFs in the CLIP assay described in (*D*). The three primer sets used for qPCR are shown on the X-axis. (*F*) Knock-down of DHX30 in WT MEFs using siRNA. (*G*) A schematic diagram of DRIP-qPCR using the S6 antibody to immunoprecipitate RNA-DNA hybrids. (*H*) Quantification of R-loops mapping to mt-tRNAs indicates that DHX30 knock-down decreases mt-tRNA R-loop resolution (n = 7). Results are shown as the mean ± SEM. *P < 0.05; **P < 0.01; ****P < 0.0001.

The R-loop, which is composed of an RNA-DNA hybrid and a displaced single-stranded complementary DNA, is an intermediate structure formed during transcription (18, 33). Failure to properly resolve the R-loop can lead to improper RNA processing. Since EndoG cleaves kinked DNA, which is present at the ends of R-loops, we hypothesized that mt-tRNA^Thr^ may be trapped in the R-loops and is not processed properly in EndoG KO liver. To test this possibility, we immunoprecipitated DNA-RNA hybrids with anti-DNA:RNA monoclonal antibody S9.6 and performed the DNA-RNA Immunoprecipitation-seq (DRIP-seq) analysis (34) of the mitochondrial genome. EndoG KO mitochondria contained significantly higher levels of R-loops that mapped to mt-tRNA^Thr^ and, surprisingly, to six other mt-tRNAs (Gly, Ser, Phe, Val, Gln, and Ala) as well as the two mt-rRNAs (12S and 16S) (Fig. 4*A*).

Resolution of R-loops in a polycistronic genome may require not only cleavage by a nuclease (e.g., EndoG) but also the unwinding of the DNA:RNA hybrid by an RNA helicase that is recruited to the cleaved site (36). The RNA helicase DHX30 drew our attention because it binds to the mitochondrial genome (37) as well as to the mtRNA near the noncoding region 3′ of mt-tRNA^Thr^ (38) and promotes mitochondrial translation (19)and OXPHOS (39). Moreover, it interacts with EndoG in a coimmunoprecipitation assay (Fig. 4*B*). To investigate the potential role of EndoG in DHX30 recruitment to mtRNA, we performed the UV-crosslinking and immunoprecipitation (CLIP) (38) assay in WT and EndoG KO MEFs by using anti-DHX30 antibody (Fig. 4 *C* and *D*). The CLIP assay shows that WT cells have more DHX30 bound to mt-RNA than EndoG KO cells (Fig. 4*E*). If EndoG recruits DHX30 to resolve R-loops, DHX30 deficiency should increase R-loop entrapment of mt-tRNAs. Indeed, DRIP-qPCR in WT MEFs revealed that knocking down DHX30 with siRNA increased mt-tRNAs (mt-tRNA^Thr^ and mt-tRNA^Val^) trapped in R-loops (Fig. 4 *F*–*H*). It is interesting that mt-tRNA^Asp^, which had background level of R-loop entrapment from DRIP-seq (Fig. 4*A*), also had higher R-loop entrapment with DHX30 knock-down. The levels of DHX30 and EndoG were not linked, because DHX30 levels were similar in the livers of EndoG WT and KO mice on HFD (*SI Appendix*, Fig. S9). Taken together, these findings suggest that EndoG collaborates with DHX30 to resolve mitochondrial R-loops.

### EndoG Cleaves the mt-tRNA^Thr^ Precursor in the MirPro Sequence.

**Fig. 5.**
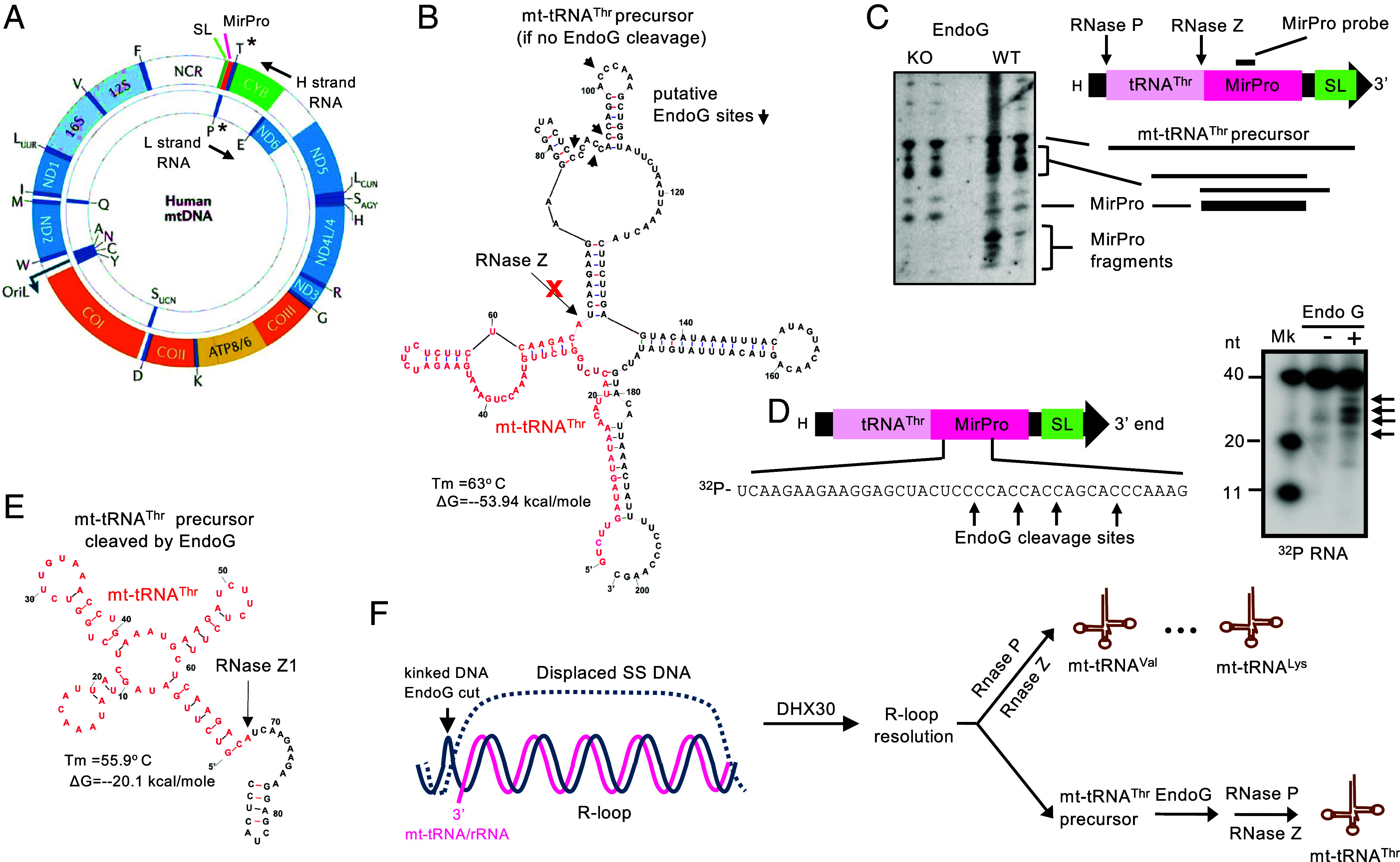
EndoG cleaves the mt-tRNA^Thr^ precursor in the MirPro sequence. (*A*) Diagram of the mitochondrial genome (42): larger circle, H-strand; smaller circle, L-strand. The directions of the transcription of the H and L strands are shown with arrows above and below each strand, respectively. Mitochondrial tRNA^Thr^ (T, on H-strand) and tRNA^Pro^ (P, on L-strand) are marked with *. (*B*) The predicted secondary structure and stability of the mt-tRNA^Thr^ precursor (IDT program). The sequence of mt-tRNA^Thr^ is colored in red font. RNase Z is unable to cleave the mt-tRNA^Thr^ precursor to generate a correct 3′-end because of its abnormal secondary structure. Putative EndoG cleavage sites in the MirPro region detected in vivo and in vitro (see panels *C* and *D*) are also shown. (*C*) (*Left*) Northern blot showing the mt-tRNA^Thr^ precursor (mt-tRNA^Thr^ + 3′ end), intermediates and fragments in EndoG WT and KO liver. (*Right*) The scheme of 3′-end of the H-strand RNA, MirPro, and the stem-loop (SL), mt-tRNA^Thr^ processing sites (RNases P and Z), and mt-tRNA fragments are indicated. The location of the MirPro probe is shown at the *Top*. (*D*) (*Left*) EndoG cleavage sites in MirPro RNA sequence are shown. (*Right*) MirPro fragments generated by incubating the 5′-^32^P labeled MirPro sequence with recombinant EndoG. (*E*) The predicted secondary structure and stability of the mt-tRNA^Thr^ precursor after it has been cleaved by EndoG. This structure can be cleaved by RNase Z. (*F*) Schematic diagram of the role of EndoG (with DHX30) in R-loop resolution of mtRNAs such as tRNA and rRNA. For R-loop resolution, EndoG cleaves the kinked DNA at the end of R-loops to allow R-loop unwinding by DHX30. This is presumably sufficient for RNases P/Z cleavage of all mt-tRNAs except mt-tRNA^Thr^. The precursor of mt-tRNA^Thr^ needs further EndoG cleavage in the MirPro region before it can be properly folded for further processing.

Since EndoG is important for the R-loop resolution of many mt-tRNAs and both mt-rRNAs (Fig. 4*A*), mt-tRNA^Thr^ must have a unique feature(s) that further requires EndoG for its production. First, it is the 3′-most gene on the H-strand polycistronic transcript (Fig. 5*A*). Second, the predicted secondary structure of the H-strand RNA 3′-end contains two strong structures immediately downstream of mt-tRNA^Thr^ that are noncoding: 1. the sequence complementary to mt-tRNA^Pro^, which is encoded on the light (L)-strand, also known as mirror mt-tRNA^Pro^ (MirPro); 2. stable stem-loop (SL) structure 3′ of MirPro (38). Noteworthy, the sequence next to SL has strong potential to form stable secondary structure by base-pairing with mt-tRNA^Thr^ 5′-part (Fig. 5*B*). This structure representing one of the most probable folds could interfere with the canonical cloverleaf-folding of mt-tRNA^Thr^ which is required for its excision by RNases P (5′) and Z (ELAC2) (3′) (40, 41). High-resolution northern blot hybridization of RNA from EndoG WT and KO livers with the probe complementary to the sequence downstream of mt-tRNA^Thr^ (annotated as MirPro probe) confirmed the presence of stable RNA corresponding to the H-strand transcript 3′-end (annotated as mt-tRNA^Thr^ precursor, Fig. 5*C*) and intermediate mtRNA fragments in both EndoG WT and KO. However, the most striking difference between them is the presence of small RNA fragments in EndoG WT livers but not in KO livers (Fig. 5*C*). We speculated that these fragments can result from RNA cleavage in the MirPro region (as shown in Fig. 5*B*). The MirPro sequence contains multiple C-rich motifs that could be cleaved by EndoG, and indeed, recombinant EndoG cleaved ^32^P 5′-labeled MirPro RNA in the C-rich motifs (Fig. 5*D*). The short 3′-extension of mt-tRNA^Thr^ after EndoG cleavage is not predicted to interfere with mt-tRNA^Thr^ cloverleaf-folding and thus the formation of its correct 3′-end required for tRNA stability and subsequent aminoacylation (Fig. 5*E*). Therefore, our data demonstrate that EndoG functions in mt-tRNA^Thr^ processing by removing the noncoding 3′-extension of the H-strand mt-RNA.

## Discussion

Our findings indicate that EndoG regulates mitochondrial function by regulating mt-tRNA^Thr^ production and therefore, intramitochondrial translation. We propose that EndoG cleaves kinked DNA at the end of R-loops allowing DHX30 helicase to unwind R-loop, freeing many mt-tRNAs for excision by RNases P/Z and further processing. However, unlike other mt-tRNAs, mt-tRNA^Thr^ is contiguous to noncoding sequences at the 3′-end of the H strand mtRNA that could form strong secondary structures that compete with its cloverleaf-folding which is required for cleavage by RNases P/Z. Therefore, we propose the “deblocker” model in which EndoG promotes the OXPHOS activity by not only resolving R-loops but by additionally cleaving MirPro and removing the 3′ sequence that could block mt-tRNA^Thr^ processing (Fig. 5 *E* and *F*). It should be noted that this conclusion is based on RNA folding algorithms, and predictions based on RNA folding algorithms are subject to error without orthologous data.

Interestingly, in mammalian mtDNA, threonine codons are among the most common, ranking just behind leucine and isoleucine, likely because of their hydrophobic properties. However, the amount of mt-tRNA^Thr^ is usually quite low under normal conditions (43). We propose that the inverse relationship between the frequent use of threonine codons and the low levels of mt-tRNA^Thr^ has a regulatory role. Even a slight further decrease in mt-tRNA^Thr^ levels could impact mitochondrial protein synthesis compared to that of other mt-tRNAs.

EndoG deficiency significantly decreased the activity of only OXPHOS complexes I and IV, most likely because complex II does not contain any proteins encoded by the mitochondrial genome and complex III contains only one, CYTB. Furthermore, only complexes I and IV are affected by mutations in mt-tRNAs in diseases such as MERRF and MELAS (44). In this respect, our work conflicts with a previous report (45) which found that EndoG WT and KO skin fibroblasts had similar OXPHOS activity and OCR. This could be attributed to different tissue sources for the fibroblasts (MEFs vs. skin fibroblasts). The metabolic differences that we find between WT and KO mitochondria in the MEFs are supported by the findings in the liver (*SI Appendix*, Fig. S2). One caveat to our conclusion drawn from MEFs is that they were derived from sex-mixed embryos. Although it is unclear whether the sexual dimorphism of MASLD extends to MEFs, we acknowledge that we cannot rule out the possibility that the differences in sex composition affected MEF metabolism.

Recently Wang et al. (9) reported that cytoplasmic EndoG increases MASLD by increasing the cytoplasmic mTORC2-AKT-ACLY signaling in females only. Their work found that male EndoG KO mice have no MASLD phenotype whereas ours do. The reason for this discrepancy is not clear, but one possibility may be that we used a different substrain of mice. The reason for the sexual dimorphism in MASLD observed in EndoG knockout mice is unclear. However, the higher incidence of MASLD in men and postmenopausal women compared to premenopausal women suggests that estrogen may offer protective effects against the disease (46). EndoG-related sexual dimorphism has also been observed in flies with deficiency of the EndoG ortholog: EndoG-depletion was protective for α-synuclein-driven neurodegeneration in female flies, but was significantly less effective in males (47).

Unlike restriction endonucleases, EndoG does not have absolute sequence specificity. However, EndoG combines its preferences for C-rich sequences and kinked secondary structure for its cleavage site specificity. Nuclease complexes such as RISC (48) and Crispr-Cas9 (49) also regulate gene expression by cleaving RNA and DNA, respectively. However, unlike EndoG, the nuclease subunits of these complexes do not have sequence specificity of their own but rely on guide RNAs to provide sequence specificity. As such, EndoG may represent a class of nucleases that regulates gene expression.

EndoG expression and activity are suppressed in MASLD (Fig. 1) and under oxidative stress conditions (50), respectively. This suggests that EndoG may function as a mitochondrial stress sensor. Since EndoG deficiency leads to MASLD in male mice, reduced levels of EndoG in MASLD may contribute to the development of MASLD. At the level of individual mitochondrion, EndoG deficiency decreases the high-OCR mitochondrial subpopulation while simultaneously increasing the low-OCR mitochondrial subpopulation. Supplementation with mt-tRNA^Thr^ largely reverses these abnormalities. Therefore, increasing hepatic EndoG activity or mt-tRNA^Thr^ levels may serve as a therapeutic strategy to ameliorate MASLD.

## Materials and Methods

### Human Liver Sample: RNA Extraction and RNA-seq.

Liver biopsies were obtained from subjects with MASLD participating in a clinical trial investigating the effects of vitamin E (ClinicalTrials.gov, NCT017921150). Subjects had compensated MASLD confirmed by ^1^H-magnetic resonance spectroscopy with no alcohol consumption. The study was approved by the NIDDK/NIAMS Institutional Review Board, and all subjects gave written informed consent. Study methods and inclusion criteria are detailed elsewhere (51). Briefly, baseline percutaneous needle liver biopsies were obtained prior to treatment, immediately placed in RNAlater (Thermo Fisher Scientific), and stored at −80 °C. RNA was extracted with Trizol (Thermo Fisher Scientific) and purified with the miRNAeasy kit (Qiagen, Germany). Extracted RNA was assessed using an Agilent 2100 Bioanalyzer for quality. PolyA-enriched RNA was used to prepare a paired-end index library. Samples were pooled (2 pools) and run on an Illumina HiSeq4000 with 75 bp paired-end sequencing.

RNA-seq was performed on 19 MASLD patients’ liver samples from 7 females and 12 males with age range: 23 to 46. The RNA sequence was subjected to quality control (FastQC, a quality control tool for high-throughput sequence data and available online at: http://www.bioinformatics.babraham.ac.uk/projects/fastqc), and trimmomatic (0.37; https://github.com/timflutre/trimmomatic) to remove adapters, followed by alignment to the human genome (GRCh38) using HISAT2.2 (https://daehwankimlab.github.io/hisat2/). Gene expression levels were quantified using featuresCounts (PMID: 24227677).

The liver RNAseq data from the GTEx (gene tissue expression consortium) were used as control. Briefly, 19 liver RNAseq data were subset from the GTEx to match the age and sex in the MASLD sample cohort described above. ComBat R package (PMID: 33015620) was used for batch correction to integrate the GTEx data with the liver-MASLD data using 4 control and 8 MASLD samples from the publicly available RNAseq data GSE130970. Differential expression analysis was performed on the batch-corrected 23 controls and 27 MASLD samples using limma voom (PMID: 24485249).

### Mouse Liver Sample: RNA Extraction and RNA-seq.

RNA from the livers from WT and EndoG KO male mice which had been on a high-fat diet for 3 mo was isolated and purified by the Qiagen RNeasy Mini Kit following the manufacturer's protocol. RNA integrity was checked using the Agilent Bioanalyzer. For RNA-seq, total ribosomal RNA was removed, and the RNA-seq libraries were constructed from 100 to 500 ng of total RNA using the TruSeq Stranded Total RNA Library Prep (#20020596; Illumina) and Ribo-Zero rRNA Removal (#MRZH11124; Illumina) kits. The fragment size of the RNA-seq libraries was verified using a 2100 Bioanalyzer instrument (#G2939BA; Agilent Technologies) and concentrations were determined using a Qubit 3 fluorometer (#Q33226; ThermoFisher Scientific). Libraries were loaded onto HiSeq 3000 (#SY-401–3001; Illumina) for 2 × 75 bp paired-end read sequencing. Fastq files were generated using bcl2fastq Conversion Software v2.20 (Illumina).

The RNA-seq data were analyzed using the RNA-Seek pipeline. The sequences were aligned to the mm10 genome with the STAR aligner, and gene counts were calculated using RSEM. Differentially expressed genes between WT and EndoG KO livers were identified using DESeq2, with the data normalized for library size. Genes were ranked based on fold change from the DESeq2 analysis, and these rankings were applied in GSEA using the classic statistical approach.

### Animals.

All experiments were approved by the NHLBI ACUC (Animal Care and Use Committee). *Ob/ob* mice in C57 background were purchased from Jackson Laboratory (000632). EndoG KO mouse was generated by Michael Lieber, University of Southern California (52). These EndoG KO mice were backcrossed to C57BL/6J (WT, Jackson Laboratory, 000664) for 13 generations to generate congenic WT and KO littermates. Mice were housed with a 12/12—light/ dark cycle with free access to food and water. Unless indicated otherwise, mice were fed NIH-31 open formula mouse/rat diet (sterilizable).

To study the effect of EndoG on diet-induced HS, male EndoG WT and KO mice were randomly assigned to regular chow diet (10 and 70% of total calorie from fat and carbohydrate, respectively, #D12450B, Research Diets Inc., New Brunswick, NJ) or HFD (45 and 35% of total calorie from fat and carbohydrate, respectively, #D12451) for 12 wk. For aging studies, mice were put on our standard facility diet (LabDiet #5021).

### Cell Culture.

MEFs and NIH3T3 cells were cultured with DMEM supplemented with 10% FBS and 1% penicillin/streptomycin in 5% CO_2_ incubator at 37 °C.

### Mitochondrial DNA Copy Number Analysis.

Total DNA was extracted from fresh liver tissues or cells using DNeasy® Blood & Tissue Kit (Qiagen), according to the manufacturer’s protocol. QPCR assay performed and analyzed on a Lightcycler® 96 Real-Time PCR System (Roche Life Science) to analyze mitochondrial DNA (mtDNA) copy number. Mitochondrial gene *nd4* was used to evaluate mtDNA copy number and nuclear gene *mTert* gene was used as internal control. Results were expressed as ratio of *nd4* to *mTert*.

### Seahorse Assay.

Cellular metabolic rates were measured using a XF24 Analyzer (Seahorse Bioscience, North Billerica) as described before. Cells were seeded in XFe24-well cell culture microplates (Seahorse Bioscience, North Billerica) at 1.0 × 10^5^ cells/well in 100 μL of growth medium (high glucose DMEM, 10% FBS, 1% penicillin/streptomycin) and allowed to rest at room temperature for 1 h in order to promote an even cell distribution. Cells were then incubated at 37 °C and 5% CO_2_ for 3 h in order to allow sufficient time for adherence to cell culture microplate. Prior to the assay the cells were washed once and placed in 500 µL of Seahorse XF Base Medium (glucose 10 mM, sodium pyruvate 1 mM, glutamine 1 mM, pH 7.4) prewarmed to 37 °C. The plate was then transferred to a non-CO_2_ incubator for 1 h. Following calibration, oxygen consumption measurements (OCR) were performed following the sequential addition of oligomycin (1 µM), carbonyl cyanide *p*-trifluoromethoxyphenylhydrazone (3 µM), and antimycin A/rotenone (1 µM). Upon completion of the assay, cells were collected in sucrose lysis buffer (50 mM Tris pH 7.5; 270 mM sucrose; 1 mM EDTA; 1 mM EGTA; 1% Triton X-100; 50 mM sodium fluoride; 5 mM sodium pyrophosphate decahydrate; 25 mM beta-glycerolphosphate; 1 complete™ protease inhibitor cocktail EDTA free tablet) and centrifuged for 10 min at 8,000 g and the supernatant was removed for protein determination. Protein concentration was determined using the DC protein assay (Bio-Rad, Hercules, CA). OCR is reported relative to protein content (pmol/min/µg).

### ROS Measurement.

To measure mitochondrial ROS level, EndoG WT and KO cells were incubated with 5 μM MitoSOX Red for 30 min in Hanks’ balanced salt solution (Life Techonologies) at 37 °C. After they were rinsed 3 times with PBS, fluorescence intensity was analyzed by flow cytometry.

CM-H2DCFDA was used to determine ROS levels in mouse liver tissues. Fresh liver tissue was weighed and homogenized with ice-cold 40 mM Tris-HCl buffer (pH 7.4). The homogenate was sonicated to further break tissues. Sonicates were further diluted to 0.25% with ice-cold TRIS buffer. Each sample was equally split into two fractions. One fraction was incubated with 25 μM CM-H2DCFDA for 15 min at 37 °C in water bath. The second fraction incubated with methanol was used as a “blank” control for tissue autofluorescence. Fluorescence intensity was measured at 488 nm excitation and 525 emmision using a Cytation 3 imaging reader.

### Palmitic Acid Treatment.

Palmitic acid was melted at 70 °C and dissolved in 0.1 M NaOH at 100 mM. Then, the palmitic acid was conjugated with 10% FFA-free BSA at 55 °C in water bath, giving a final concentration of 5 mM. After filtration, the palmitic acid/BSA stock solution was stored at −20 °C until used. BSA solution (10%) was used as the control. EndoG WT and KO MEF cells were treated with 0.3 mM palmitic acid overnight. Live and dead cell number were measured by with Countness II aUTOMATED cell Counter (Life Tech).

### Lipid Accumulation, Visualization, and Quantification.

After MEF cells were challenged with 0.5 mM Oleic acid (Sigma-Aldrich) overnight, they were fixed with 4% paraformaldehyde for 15 min at room temperature. The fixed cells were washed with PBS 3 times and then stained with BODIPY (1 mg/mL) for 30 min in the dark. DAPI was used to stain nuclei. Subsequently, the cells were rinsed with PBS 3 times and mounted on a glass slide for visualization with a confocal microscope.

To quantify lipid accumulation, live MEF cells were incubated with BODIPY for 30 min in the dark after they were treated with 0.5 mM Oleic acid (Sigma-Aldrich) overnight. After they were washed with PBS 3 times, BODIPY immunofluorescence was quantified with flow cytometry.

### Serum Chemistry.

After mice were fasted overnight, blood was collected into an EDTA-coated Eppendorf tube. The blood sample was centrifuged at 3,000 rpm for 15 min at 4 °C, supernatant (plasma) was collected for biochemical analysis by the Department of Laboratory Medicine at the NIH Clinical Center.

### Histological Examination for Lipid Droplet Accumulation.

Hepatic lipid droplet accumulation was examined by hematoxylin and eosin (H&E) and Oil Red O staining. Livers were harvested after the mice were killed under adequate anesthesia with CO_2_. Livers were fixed in 10% neutral-buffered formalin for 24 h at room temperature. After dehydration with serial alcohols and clear with xylenes, the specimen was embedded in paraffin for sectioning at 10 μm. H&E staining was performed to examine lipid accumulation on the sections. For Oil Red O staining, livers were collected and snap-frozen in isopentane-cooled liquid nitrogen before being sectioned at 10 μm with a cryostat. After being fixed with 4% paraformaldehyde and treated with absolute propylene glycol, Oil Red O staining was performed to visualize lipid droplets in the liver. Hamamatsu nanozoomer was used to digitalize sections.

### Hepatic Triglyceride Measurement.

Frozen liver tissues were used to measure hepatic triglyceride. After being homogenized in 5% Triton X-100, hepatic samples were incubated in a water bath at 90 °C for 5 min. Subsequently, samples were cooled down at room temperature followed by centrifugation for 2 min at room temperature. Supernatant was collected and diluted for triglyceride analysis with the triglyceride colorimetric assay kit (Biovision, #K622-100).

### Isolation of Mitochondria from Livers and Cells.

Mitochondria were isolated as previously described. Mitochondrial isolation buffer (225 mM mannitol, 75 mM sucrose, 5 mM MOPS, 0.5 mM EGTA, and 2 mM taurine (pH 7.25) supplemented with protease inhibitor cocktail (Roche) was prepared freshly and placed on ice. Fresh liver tissue was weighed (0.3 g) and then minced finely with scissors in 1.5 mL mitochondrial isolation buffer on ice. Next, the sample was transferred into a prechilled 2.0 mL Dounce tissue homogenizer. On ice, the sample was homogenized with pestle A for 15 strokes followed by pestle B for 25 strokes. To isolate mitochondria from cells, cell pellets resuspended in mitochondrial isolation buffer were directly transferred into the Dounce homogenizer and homogenized with pestle B. The homogenate was then centrifuged at 600 g for 5 min at 4 °C. Then the supernatant was collected and centrifuged again at 600 g for 5 min at 4 °C. Subsequently, the supernatant was centrifuged at 11,000 g for 6 min at 4 °C and mitochondrial pellet was collected for further experiment.

### Mitochondrial DNA Sequencing.

Mitochondria were isolated from the livers of 12-mo-old EndoG WT and KO mice as described previously (53). After extracting DNA from mitochondria, high-throughput DNA sequencing was performed by following the Illumina HiSeq 2000 standard protocol. The raw reads (5.8 to 7.3 million reads) in fastq format were mapped to mm10 mouse chromosome M reference sequence by BWA (0.7.10-r789) with mem algorithm. SAMtools (version 0.1.19) removed the low-quality reads (MAPQ < 30) and PCR duplication reads. All samples have over 7,800× average coverage on chrM. The insert sizes of the paired-reads were retrieved from the mapped sam files. The mutation frequency was computed on each base from the output of samtools mpileup at each position. The SNPs mutation rates and the inner sizes between two groups’ samples were evaluated by data distribution and nonparametric test. We also applied BreakDancer software to detect insertion, deletion, or intrachromosomal translocation between two condition groups.

### tRNA-seq.

Arraystar Inc. performed tRNA-seq using total RNA from young (2-mo-old) mice liver tissues. First, the samples were treated with 0.1 M Tris-HCl and AlkB to deacylate 3′-aminoacyl and demethylate m1A and m3C, respectively. After reverse transcription, ~180 to 210 bp PCR amplified fragments were extracted and purified from the PAGE gel. The prepared tRNA-seq libraries were finally quantified using Agilent 2100 Bioanalyzer, then sequenced using Illumina Hiseq system.

### qRT-PCR.

Total RNA was extracted from young (2.5-mo-old) mice liver tissues with Trizol reagent (Invitrogen) or cells with the RNAeasy Mini Kit (Qiagen). After DNA was removed with Turbo DNA-free DNase treatment (Ambion), the diluted RNA was reverse transcribed by using SuperScript® III First-Strand Synthesis system (Invitrogen). Quantitative real-time PR-PCR was performed and analyzed on a Lightcycler® 96 Real-Time PCR System (Roche Life Science). *Gapdh* mRNA was used for normalization.

**Table:** 

Gapdh F:	GACTTCAACAGCAACTCCCAC,
R:	TCCACCACCCTGTTGCTGTA
ATP8 F:	CAAACATTCCCACTGGCACC
R:	TTGTTGGGGTAATGAATGAGGCA
16S F:	GTTACCCTAGGGATAACAGCGC
R:	GATCCAACATCGAGGTCGTAAACC
COX2 F:	CACTCATGAGCAGTCCCCTC
R:	ACCCTGGTCGGTTTGATGTT
ND4L F:	ATGCCATCTACCTTCTTCAA
R:	AAACTAAGGTGATGGGGATT
CYTB F:	AGTAGACAAAGCCACCTTGA
R:	CCGCGATAATAAATGGTAAG
ND2 F:	CGTCACACAAGCAACAGCCTCAAT
R:	TGTGCAGTGGGATCCCTTGAGTTA
COX1 F:	GCCCCAGATATAGCATTCCC
R:	GTTCATCCTGTTCCTGCTCC
ND6 F:	TTAGCATTAAAGCCTTCACC
R:	CCAACAAACCCACTAACAAT
ND4 F:	AATCGCCTACTCCTCAGTTAGCCA
R:	AGGAGTGATGATGTGAGGCCATGT
ND5 F:	ATAGCCTGGCAGACGAACAAGACA
R:	AATTAGTAGGGCTCAGGCGTTGGT
ND1 F:	CAGGATGAGCCTCAAACTCC
R:	GGTCAGGCTGGCAGAAGTAA
ATP6 F:	AATTACAGGCTTCCGACACAAAC
R:	TGGAATTAGTGAAATTGGAGTTCCT
COX3 F:	GAAGCCGCAGCATGATACTG
R:	TTTTTTTTTTTTTTTTTTTTTTTTTAAGATC
ND6 F:	GTTGGAGTTATGTTGGAAGGAGGG
R:	CCGCAAACAAAGATCACCCAGCTA
ND3 F:	CCGCAGCATGATACTGACAT
R:	TGAATTGCTCATGGTAGTGGA

### RNA Analysis by Northern Blot Hybridization.

RNA was isolated from liver tissue, MEF cells, or mitochondria by TRIzol extraction, separated by 8% urea PAGE, electroblotted on Hybond N membrane (Amersham), and hybridized with 5’-P^32^-labeled oligonucleotide probes as previously described (54). Hybridization signals were revealed by “Typhoon TRIO” (GE Healthcare) and quantified using the *ImageQuantTL* software.

Hybridization probes:

Mt-tRNA^Pro^ mouse: CTA CTC CCC ACC ACC AGC ACC C
  Mt-tRNA^Thr^ mouse: GGTGTCTTGAGAAGAGAAGATC
  Mt-tRNA^Thr^ human: GGTGTCCTTGGAAAAAGG
  Mt-tRNA^Lys^ mouse: GGTGACTATGGAGATTTTAAGG
  Mt-tRNA^Val^ mouse: GGGATCCTGGTCAGAGTGTTCATTGGTC
  MirPro (15396-15366): GCTTTGGGTGCTGGTGGTGGGGAGTAGCTCC
  rRNA 5S: AAGTAAGCACTGTTTCAG

### Mitochondrial Translation Assay.

Analysis of mitochondrial protein synthesis was performed as previously described (32) with minor modifications. Briefly, 600 × 10^3^ cells were incubated for 10 min in DMEM w/o methionine (Sigma) in the presence of 100 μg/mL of emetine to inhibit cytoplasmic translation, followed by 30 min incubation with 200 μCi/mL [^35^S]-methionine (Amersham, 400 Ci/mmol), and, finally, 10 min chase in the normal growth medium. Cells were solubilized in a Laemmli’s buffer, sonicated, and 100 μg of protein were run on a 15% SDS-PAGE and electroblotted on nitrocellulose membrane Protran (Amersham). Dried blot was exposed with a PhosphorImager plate, visualized using “Typhoon TRIO” and quantified by *ImageQuantTL* software (GE Healthcare). After this, western immunoblotting was performed.

### Transcription of Mitochondria tRNAs.

Mitochondrial tRNA genes were amplified from total DNA of mouse or human cells and cloned into pUC19 vector under T7 promoter. Plasmids were cut by Mva I to create a CCA sequence at 3′-end and used as templates for in vitro T7 transcription by the T7 RiboMax kit (Promega). The transcripts were purified by 10% urea PAGE, eluted, and ethanol precipitated.

Mt tRNA sequence (without 3′-end CCA)

C57BL/6J mt tRNA^Thr^: GTCTTGATAGTATAAACATTACTCTGGTCTTGTAAACCTGAAATGAAGATCTTCTCTTCTCAAGACA

C57BL/6J mt tRNA^Lys^:

CACTATGAAGCTAAGAGCGTTAACCTTTTAAGTTAAAGTTAGAGACCTTAAAATCTCCATAGTGA

Primers for cloning:

Thr T7 Mus C57BL/6J: GGGAAGCTTTTTTTAATACGACTCACTATAGTCTTGATAGTATAAAC

mt tRNA^Thr^ CCA C57BL/6J: GGGATCCTGG TGT CTT GAG AAG AGAAG

Lys T7 Mus C57BL/6J: GGGAAGCTTTTTTTAATACGACTCACTATAGACTATGAAGC TAAGA GC

mt tRNA^Lys^ CCA C57BL/6J: GGGATCCTGGTGACTATGGAGATTTTAAGG

### Transfection of mt-tRNA.

Transfection of cultured cells with T7-transcripts of mt tRNAs was performed using Lipofectamine2000 (Invitrogen), basing on previously described protocol (54). Briefly, 1 μg of T7-transcript and 12.5 μL of Lipofectamine2000 was used for transfection of 2*106 cells. Cells were seeded 1 d prior transfection in order to obtain approximately 80% of confluence at the moment of transfection. T7-transcripts and Lipofectamine2000 were diluted each in 625 μL of OptiMEM, incubated for 5 min at room temperature, mixed, and then incubated for 20 min more. RNA-lipofectamin mix was then added to cells in 3.5 mL of OptiMEM. Cells were exposed to RNA–lipofectamine complexes for 5 h, after media was changed, and cells were analyzed 24 to 48 h posttransfection.

### Tissue, Cellular, and Mitochondrial Lysate Preparation.

Frozen liver tissue fragments were transferred to a clean prechilled mortar and ground finely with a pestle in liquid nitrogen. Protein was then extracted from the ground samples with RIPA buffer supplemented with phosphatase and protease inhibitors. To extract protein from cells or mitochondrial fraction, RIPA buffer with phosphatase and protease inhibitors was directly added into the cell or mitochondrial pellet followed by pipetting up and down 20 times. Subsequently, lysates were sonicated to further break the tissues and shear DNA. After being centrifuged at 15,000 rpm for 15 min at 4 °C, supernatants were collected for protein expression analysis with Western Blot.

### Immunoblotting.

The lysate containing equal amount of protein was subjected to SDS-PAGE and the proteins were transferred to nitrocellulose membranes, incubated overnight with primary antibody at 4 °C. After the membrane was incubated with horseradish peroxidase (HRP)-conjugated secondary antibody for 1 h at room temperature, signal was visualized using Amersham Imager 600 (GE Healthcare Life Sciences). The intensity of immunoblot bands was quantified using ImageJ. The primary antibodies used are EndoG (Ab76122, Abcam), DHX30 (Ab85687, Abcam), GAPDH (5174, Cell Signaling), β-actin (4970, Cell Signaling, or (C-11) Santa Cruz Biotechnology sc-1615), PNPase/PNPT1 (Ab9617, Abcam), Rhodanese/TST1 (sc-21237, Santa Cruz Biotechnology), MT-CO1/COX1 (459600, Invitrogen), MT-CO2/COX2 (K-20) (sc-23984, Santa Cruz Biotechnology). Antibodies ND1 and ND5 are polyclonal rabbit antibodies kindly provided by Anne Lombès, INSERM, France.

### FLIM Assay.

#### Transfection of EndoG WT and KO cells with mtMyoglobin-mCherry oxygen probe.

WT and EndoG^K0^ cells were plated in μ-Slide 4-well or 8-well chambers (Ibidi GmbH, Martinsried, Germany) with a density of 4 × 10^4^ cells/chamber (70 to 90% confluent at the time of transfection). For the measurement of the mitochondrial pO_2_ level, cells were transfected with a FLIM-FRET-based O_2_ sensor, the so-called mtMyoglobin (Myo)-mCherry (55, 56). Briefly, mtMyo-mCherry plasmid DNA diluted in 1,000 µL of Opti-MEM^®^ medium (Gibco) was combined with 3 µL of Lipofectamine^®^ 2000 (Invitrogen, Carlsbad, CA) transfection reagent diluted in 1,000 µL of Opti-MEM. After a 15-min period, the DNA-Lipofectamine transfection complex was added to the cells with a final plasmid concentration of 1.5 ng/µL. After 32 h incubation at 37 °C and 5% CO_2_, the transfection media was removed, and the cells were washed with Phosphate-Buffered Saline (PBS, Gibco). The cells were then covered with 400 µL of fresh modified DMEM.

#### Treatment of the cells with Rotenone.

For inhibition of mitochondrial O_2_ consumption, cells transfected with mtMyo-mCherry were treated with 1 μM Rotenone in DMEM.

#### FLIM setup.

Two-photon FLIM was performed using an Olympus IX81 inverted confocal laser scanning microscope (Melville, NY) equipped with a tunable Mai Tai DeepSee femtosecond laser (Spectra-Physics, Santa Clara, CA) operating at 80 MHz, with wavelengths set to 850, 780, or 740 nm for the excitation of FAD, mCherry, or NAD(P)H, respectively. The excitation light was passed through a 690 nm dichroic mirror and directed to an Olympus UPLANSAPO 60×, 1.2 NA water immersion microscope objective. The laser power at the objective was kept below 18 mW to avoid photobleaching of the samples during the collection time required for FLIM. The emission was collected through the same objective, directed to the side port of the microscope (nondescanned detection), and passed through a 675 nm short pass filter to reduce scattering from the laser. The signals from FAD, mCherry, and NAD(P)H were filtered through a 552/57 nm, a 647/57 nm, or a 460/60 nm bandpass filters (Semrock BrightLine®, Rochester, NY), respectively. The filtered signals were focused on a PMC100 cooled detector and the electrical pulse output from the detector was directed into an SPC-150 photon counting card (both from Becker & Hickl GmbH, Berlin, Germany). The signals were synchronized with the pulses from the laser to allow for time-correlated single photon counting (TCSPC). Synchronization with the pixel, line, and frame clock from the scanning unit of the microscope was used for image registration in TCSPC mode. The cells were imaged for 40 to 80 s (depending on the brightness) to accumulate an adequate number of photons per pixel for further analysis. Image size was set to 256 × 256 (pixels)^2^, and TCSPC histograms were collected with 256 channels in a 12.5 ns time window.

To provide an environment that is nonperturbing and suitable for the homeostasis of the cells during imaging, a miniature incubator chamber connected to a gas mixing system (Bioscience Tools, San Diego, CA) was mounted on the microscope stage. The incubator provided temperature control and the gas mixing system delivered preset mixtures of N_2_, O_2_, and CO_2_ inside the chamber. For O_2_ depletion, experiments were conducted by blanketing the cultures with different humidified mixtures of N_2_ and O_2_ with 5% CO_2_ (containing ≤ 10 ppm O_2_; AirGas) at 37 °C. Our system typically allows the cell culture (in culture dishes with medium ~1 to 2 mm deep, without lids) to equilibrate to a precise O_2_ concentration ([O_2_]) within 45 min. Measurements of oxygen partial pressure (pO_2_ in mmHg) in the culture media were performed by using a bare-fiber oxygen sensor connected to an OxyLite Pro 2 Channel monitor (Optronix Ltd., Oxford, UK). The sensing volume is a hemisphere ~250 µm in diameter on the fiber tip that therefore averages media pO_2_ from the dish surface to ~120 µm above.

#### Fluorescence lifetime analyses.

Fluorescence lifetime decay images were analyzed using the software SPCImage (Becker & Hickl GmbH, Berlin, Germany). The decay curves at each pixel were fit using a nonlinear least-squares method to approximate a double-exponential decay model. The lifetime decays of mCherry (in the presence or absence of the FRET acceptor myoglobin), NAD(P)H and FAD were also obtained by a multiexponential model in SPCImage to optimize goodness of fit (*χ*^2^). A number of parameters including short (*τ*_1_) and long (*τ*_2_) lifetimes, pre-exponential factors (*a*_1_% and *a*_2_%), and average lifetime (*τ*_mean_) were generated via amplitude weighting for each pixel; *a*_1_ and *a*_2_ can be used (if natural lifetime is constant) to represent the fraction of fluorophores with shorter and longer lifetimes, respectively. In order to compare the glycolysis and oxidative phosphorylation (OXPHOS) metabolism, the FLIM-based redox ratio (FLIRR) (57) was calculated using the ratio of bound NAD(P)H/bound FAD (*a*_2,NAD(P)H_ %/*a*_1,FAD_ %) for both WT and EndoG^K0^ cells. Finally, color-mapped lifetime images of mtMyo-mCherry, free/bound distribution of NAD(P)H (*a*_1_%/*a*_2_%), and FLIRR in the intracellular environment were obtained for each cell.

#### Analyses of FLIM data for estimation of the intracellular pO_2_.

Full image averaged values of mtMyo-mCherry fluorescence lifetime (*τ*([pO_2_])), taken for multiple cells and loci, were correlated with imposed pO_2_ by the best hyperbolic curve for the data using the Curve Fitting Toolbox in MATLAB R2016b (The MathWorks Inc., Natick, MA) (5):

[1] $$\tau \left({\mathrm{pO}}_{2}\right)=\left(\tau_{max}-0.914\right)\times \left({\mathrm{pO}}_{2}/\left(K+{\mathrm{pO}}_{2}\right)\right)+0.914,$$

where *K* is a fitting parameter related to Myoglobin affinity and *τ*_max_ and *τ*_min_ are the measured lifetimes at the highest and lowest imposed pO_2_, respectively.

To obtain intracellular pO_2_,*τ*(pO_2_) responses in WT and EndoG^K0^ cells were compared to those measured for cells treated with rotenone. Since mitochondria treated with rotenone are incapable of significant O_2_ consumption, intracellular pO_2_ is assumed in this case to be equivalent to the media-imposed pO_2_. Therefore, the *τ*(pO_2_) values for the treated cells can be used as a reference for the lifetime of the probe at the environmental level of pO_2_ present in solution. Rearranging Eq. **1**, it is possible to backcalculate the effective pO_2_ at each lifetime value, fixing the *K* and *τ*_max_ to the values obtained from the rotenone data. More detailed explanation can be found elsewhere (5).

### DRIP-seq.

#### Nucleic acid isolation from mitochondria-enriched pellets.

Mitochondria were isolated from the liver of two wild type and two EndoG knock-out animals. Mitochondrion-enriched samples were resuspended with freshly made 1% formaldehyde in phosphate-buffered saline (PBS, 136 mM NaCl, 2.6 mM KCl, 12.4 mM Na_2_HPO_4_, 1.7 mM KH_2_PO_4_, pH = 7.4) supplemented with 5 mM EDTA. Mitochondria were fixed for 10 min at room temperature, with shaking (Biosan TS-100 shake; Biosan, Riga, Latvia). Formaldehyde fixation was quenched with the addition of glycine to a final concentration of 0.667 M for 5 min at room temperature. Samples were then centrifuged at 10,000×g for 10 min at 4 °C using Eppendorf 5424 centrifuge (Eppendorf, Hamburg Germany). Supernatants were discarded, and pellets were washed in PBS. Lysis was performed using a DRIP-lysis buffer (50 mM TRIS-HCl, 10 mM EDTA, 1% SDS, and 0.333 mg/μL Proteinase K (Bioline, Alexandria, NSW, Australia) at 37 °C, overnight. Lysates were extracted in phase-lock tubes (5 Prime GmbH, Hilden, Germany) by phenol (50%)-chloroform (48%)-isoamylalcohol (2%), and the nucleic acid content was precipitated by 2.5 V 100% ethanol and 10% 3.5 M sodium acetate at −20 °C for 45 min. After centrifugation with 20,000×g for 20 min at 5 °C, pellets were washed with 70 V/V% ethanol and centrifuged again for 5 min at 20,000×g. Nucleic acid pellets were dissolved in 10 mM TRIS-Cl pH 7.5. DNA concentration was measured by a NanoDrop-1000 photometer (Thermo Scientific, Waltham, MA).

#### Mitochondrial DNA-RNA hybrid immunoprecipitation (mito-DRIP).

Isolated nucleic acid samples were fragmented by sonication (Bioruptur Plus; Diagenode, Belgium; 10 cycles: 30 s “on” followed by 30 s “off”) to an average fragment size of 300 to 500 bp. 2% of the sonicated material was kept as “input.” The remaining samples were divided into two aliquots that were or were not treated with *Escherichia coli* RNaseH New England Biolabs, Ipswich, MA). RNaseH digestion (was performed at 37 °C, overnight by adding 7U of enzyme for 1 μg of measured DNA content. RNaseH-treated samples were then PCR cleanup purified (Macherey-Nagel, Düren, Germany) and eluted in 5 mM TRIS-Cl pH 7.5 solution.

DNA-RNA hybrid immunoprecipitation was performed as described in ref. 58, using the S9.6 mouse monoclonal antibody (HB-8730, ATCC) (59). Nucleic acid content of the IP and input samples was measured by a Qubit fluorometer (Thermo Fisher Scientific).

#### Library preparation and Illumina sequencing.

DRIP “IP” and “input” samples and RNaseH1-treated (negative control) DRIP samples were concentrated to less than 30 μL volume with Eppendorf Concentrator Plus (Eppendorf, Hamburg Germany) at 30 °C for 30 min. Illumina compatible sequencing libraries were generated from fragmented 1 to 10 ng of dsDNA using Ovation**®** Ultralow System V2 (Nugen, San Carlos, CA) library preparation kit according to the manufacturer’s protocol. Briefly, fragmented dsDNA samples were end-repaired and A-tailed, then Illumina compatible indexed adaptors were ligated. Adaptor ligated dsDNA were amplified, and the products were purified using MagSi-DNA NGS*^PREP^* Plus (MagnaMedics GmbH, Aachen, Germany) magnetic beads. Sequencing libraries were quantified using the Qubit fluorometer (Thermo Fisher Scientific), and the fragment size distributions were analyzed on Agilent BioAnalyzer 2100 using DNA High Sensitivity assay (Agilent Technologies, Santa Clara, CA). Sequencing libraries were normalized to the same molar concentration and pooled together. Library pool was sequenced on NextSeq500 instrument (Illumina, San Diego, CA) generating single read 75 bp long sequencing reads. Fastq files were generated automatically after the sequencing run by Illumina BaseSapce. The library preparations and the sequencing run were performed by Genomic Medicine and Bioinformatics Core Facility of Department of Biochemistry and Molecular Biology, Faculty of Medicine, University of Debrecen, Hungary.

Raw reads were aligned to the M. musculus mitochondrial reference genome (NCBI Reference Sequence: NC_005089.1) using the default parameters of Burrows-Wheeler Aligner (BWA MEM) algorithm (10). Low mapping quality reads (MAPQ < 10) and PCR duplicates were removed. Processed alignments were subjected to bamCoverage (11) to generate signal files. RPKM (Reads Per Kilobase Million) values were calculated for 20 bp binds for each sample and smoothed using a 45 bp sliding window (--binSize 15 –smoothLength 45 –normalizeUsing RPKM). The generated signal files were visualized in R 3.4.2, using the ggplot2 (https://ggplot2.tidyverse.org) and ggbio (12).

#### qPCR validation of mito-DRIP.

DRIP IP samples were diluted 10-fold in 10 mM Tris-Cl, while input samples were diluted 100-fold. The qPCR mastermix [Light cycle green 480 (Roche, Mannheim, Germany), ROX fluorescent dye (Thermo Scientific, Waltham, MA) was prepared for each well as 5 μL Light cycle green 480 MM, 0.25 μL forward primer, 0.25 μL reverse primer, 0.05 μL ROX, 0.45 μL nuclease-free water (NFW)]. Samples were measured in triplicates. No-template controls for each primer pair were prepared by substituting the 4 μL diluted sample with 4 μL NFW. The PCR was carried out in a Quantstudio 12 K Flex QPCR machine (Life Technologies, Carlsbad, CA). PCR primers were designed for the mouse mitochondrial genome by Primer3Plus. DRIP enrichment was calculated by the 2^ΔCt^ method with sample-dilution correction. Enrichment values were expressed as IP/input ratios or were normalized to RNAseH1-treated IP/input values.

DRIP-qPCR oligos:

**Table:** 

Mito71-314:	FW AAGGTTTGGTCCTGGCCTTA
	RV GGCTGGCACGAAATTTACCA
tRNA Thr:	FW TATCATTGGCCAACTAGCCTCC
	RV GGGAGTAGCTCCTTCTTCTTGATG
tRNA Val:	FW TAAAGCATCTGGCCTACACCC
	RV TCTTTCCCTTGCGGTACTAGTTC
tRNA Asp:	FW CACACATTCGAGGAACCAACC
	RV GTTGGAATGGGTAGGCCATATAAG

All chemicals were purchased from Sigma-Aldrich (owned by Merck, Saint Louis, MO) unless stated otherwise.

#### Mitochondrial complex activity.

For the respiratory complex activity measurements, mitochondria were isolated from WT and EndoG KO MEF by differential centrifugation and then disrupted by three cycles of freeze-thawing in hypotonic buffer. Spectrophotometric assays of the respiratory chain were performed by standardized protocols as described previously (60, 61), using DU730 UV/Vis Spectrophotometer (Beckman Coulter). Briefly, for Complex I, the rotenone-sensible NADH:ubiquinone oxidoreductase activity was measured by the kinetics of decreasing NADH absorbance at 340 nm. For Complex II, the succinate dehydrogenase activity was measured by following the reduction of 2,6-dichlorophenol-indophenol (DCPIP) by the decrease in absorbance of oxidized DCPIP at 600 nm. For Complex III, the antimycin A-sensitive activity of decylubiquinol cytochrome c oxidoreductase was followed by the cytochrome c reduction (increase in absorbance at 550 nm). For Complex IV, the cytochrome c oxidase activity was assessed by the disappearance of reduced cytochrome c, which absorbs at 550 nm.

#### Endonuclease G cleavage assay.

RNA oligonucleotides including MirPro and size markers were end-labeled using T4 polynucleotide kinase and γ^32^-P ATP (Perkin Elmer) and were purified using Centri-Sep spin columns (Invitrogen). For the RNA/DNA hybrids, 20 pmol each of complementary oligonucleotides were heated to 94 °C in a final volume of 45 μL of hybridization buffer (10 mM Tris, 1 mM EDTA, and 50 mM sodium chloride, pH 7.5), and then allowed to cool to room temperature.

MirPro RNA oligonucleotide: 5′-UCAAGAAGAAGGAGCUACUCCCCACCACCAGCACCCAAAG.

For the production of GST-tagged bovine EndoG in mammalian cells, HEK-293 cells (3–14 maxi-dishes/transfection) cultured at 37 °C in a humidified atmosphere of 5% CO2 in DMEM with 10% (v/v) fetal calf serum, 100 units of penicillin and 100 μg/ml of streptomycin were transfected with 15 μg of an appropriate expression construct using Transfast™ transfection reagent (Promega) as described by the supplier’s recommendations. GST-bovine EndoG was purified by GST-affinity chromatography using low-salt buffers. Radioactively labeled RNA substrate (0.8 pmol) was then digested with 0.1 to 1 μL of purified recombinant GST-bovine EndoG at 37 °C in EG buffer (50 mM sodium phosphate pH 6; 25 mM magnesium chloride; 0.01% Tween 20) in a final volume of 20 μL. Aliquots were removed at various times and the reaction was stopped by the addition of 2× urea loading buffer on ice. Aliquots were run at 25 W on 15% acrylamide/8 M urea/TBE gels and analyzed using a Typhoon FLA 7000 phosphor imager.

#### RNA secondary structure prediction.

RNA folding and structure stability predictions were done by RNAfold software (ViennaRNA Web Service) and IDT OligoAnalyzer Tool (Integrated DNA Technologies).

#### Statistical analysis.

The data are presented as the mean ± SD. Mann–Whitney tests were used to evaluate whether the lifetime values in each two independent groups (e.g., EndoG WT and KO cells and Rotenone-treated cells) measured at the same imposed pO_2_ are significantly different from each other. The data are presented as the mean ± SEM (except for FLIM). Statistical significance was determined by the multiple unpaired *t* test. Lifetime imaging was conducted for at least 20 cells for each condition. Analyses were carried out using SPSS 14.0 (a subsidiary of IBM, Chicago, IL) software, and statistical significance was defined at *P* < 0.05 (95% confidence level).

## Supplementary Material

Appendix 01 (PDF)

## Data Availability

All study data are included in the article and/or SI Appendix.
