# Supplementary material for: Endonuclease G promotes hepatic mitochondrial respiration by selectively increasing mitochondrial tRNAThr production
Source: Proc Natl Acad Sci U S A. 2025 Jan 3;122(1):e2411298122. doi: 10.1073/pnas.2411298122 (PMC11725929; doi:10.1073/pnas.2411298122)
Supplement: Supplementary file 1 — Appendix 01 (PDF) [file pnas.2411298122.sapp.pdf]

SUPPLEMENTAL FIGURES

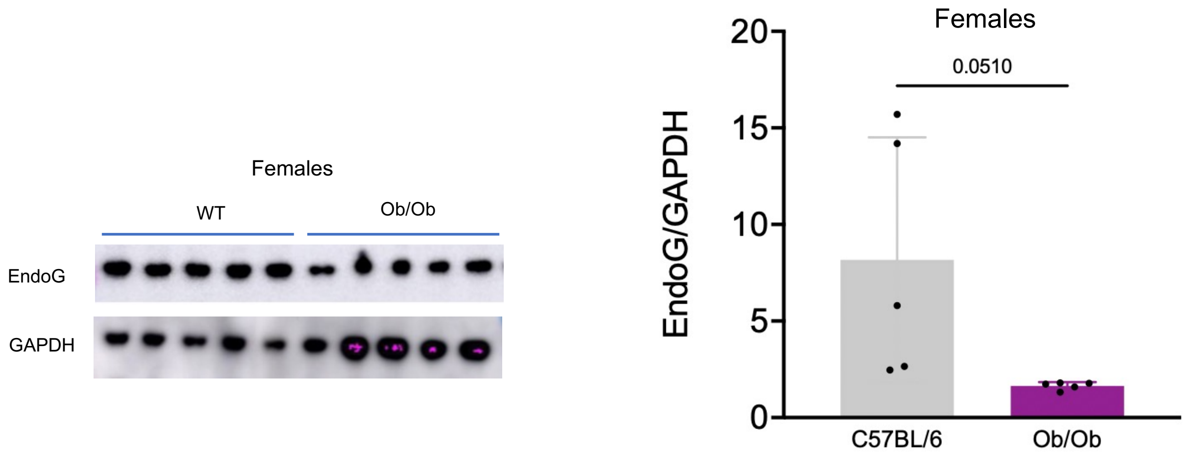

Fig. S1 EndoG expression level is decreased in female *ob/ob* liver. (Left) Protein levels of EndoG and GAPDH in female WT and *ob/ob* mice. (Right) Quantification of EndoG levels normalized to GAPDH levels.

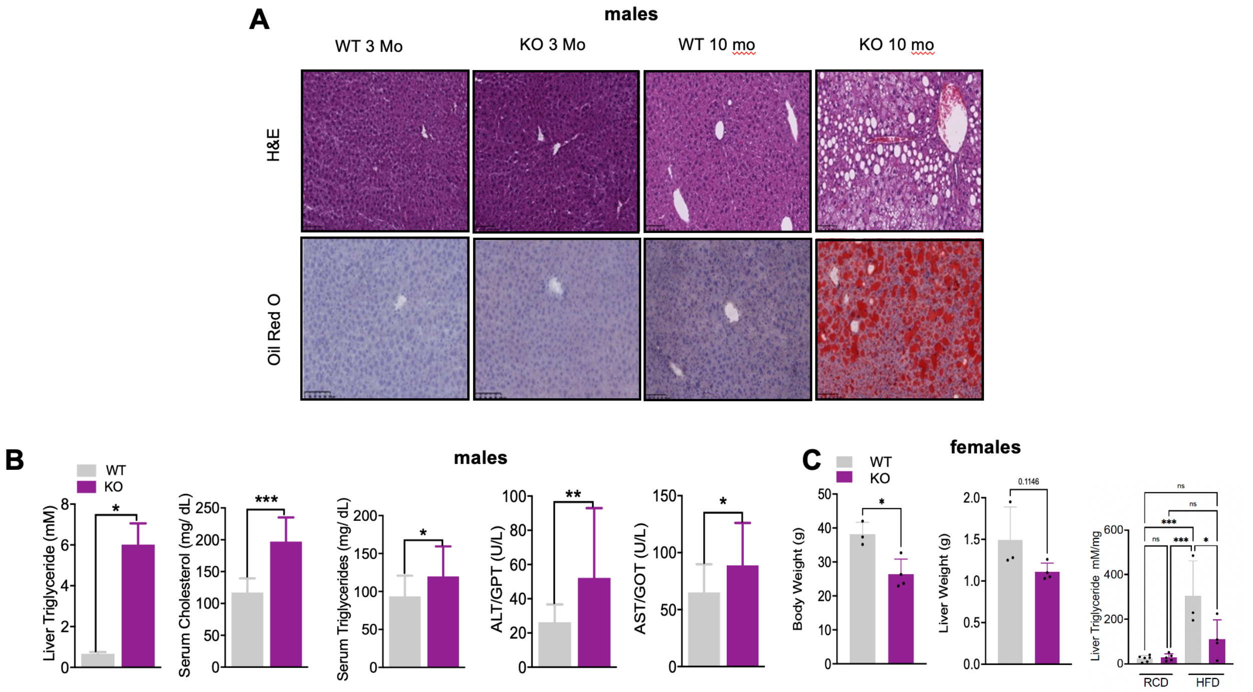

D

NAME	GS<br>follow link to MSigDB	GS DETAILS	SIZE	ES	NES	NOM p-val	FDR q-val	FWER p-val	RANK AT MAX	LEADING EDGE
HALLMARK_OXIDATIVE_PHOSPHORYLATION	HALLMARK_OXIDATIVE_PHOSPHORYLATION	Details ...	137	0.56214744	0.56214744	0	0	0	5266	tags=88%, list=32%, signal=127%
HALLMARK_BILE_ACID_METABOLISM	HALLMARK_BILE_ACID_METABOLISM	Details ...	75	0.4573716	0.4573716	0	0	0	7024	tags=88%, list=42%, signal=152%
HALLMARK_FATTY_ACID_METABOLISM	HALLMARK_FATTY_ACID_METABOLISM	Details ...	117	0.44182062	0.44182062	0	0	0	4194	tags=69%, list=25%, signal=92%
HALLMARK_CHOLESTEROL_HOMEOSTASIS	HALLMARK_CHOLESTEROL_HOMEOSTASIS	Details ...	60	0.43688694	0.43688694	0	0	0	3826	tags=67%, list=23%, signal=86%
HALLMARK_XENOBIOTIC_METABOLISM	HALLMARK_XENOBIOTIC_METABOLISM	Details ...	151	0.40108368	0.40108368	0	1.67E-04	0.001	4270	tags=66%, list=26%, signal=88%
HALLMARK_ADIPOGENESIS	HALLMARK_ADIPOGENESIS	Details ...	162	0.36788562	0.36788562	0	4.89E-04	0.003	4286	tags=62%, list=26%, signal=83%
HALLMARK_PEROXISOME	HALLMARK_PEROXISOME	Details ...	72	0.34231234	0.34231234	0	0.00114937	0.008	6078	tags=71%, list=37%, signal=111%
HALLMARK_COAGULATION	HALLMARK_COAGULATION	Details ...	106	0.29396766	0.29396766	0	0.00596711	0.047	2970	tags=47%, list=18%, signal=57%
HALLMARK_REACTIVE_OXIGEN_SPECIES_PATHWAY	HALLMARK_REACTIVE_OXIGEN_SPECIES_PATHWAY	Details ...	46	0.25986663	0.25986663	0.004	0.01464261	0.124	3983	tags=50%, list=24%, signal=66%
HALLMARK_MYC_TARGETS_V1	HALLMARK_MYC_TARGETS_V1	Details ...	161	0.24641661	0.24641661	0	0.01997221	0.185	9627	tags=83%, list=58%, signal=196%
HALLMARK_UNFOLDED_PROTEIN_RESPONSE	HALLMARK_UNFOLDED_PROTEIN_RESPONSE	Details ...	84	0.23710781	0.23710781	0	0.02336236	0.235	10078	tags=85%, list=61%, signal=215%
HALLMARK_ESTROGEN_RESPONSE_LATE	HALLMARK_ESTROGEN_RESPONSE_LATE	Details ...	121	0.22184645	0.22184645	0	0.03156254	0.324	2918	tags=40%, list=18%, signal=48%
HALLMARK_MYC_TARGETS_V2	HALLMARK_MYC_TARGETS_V2	Details ...	49	0.21482277	0.21482277	0.02357564	0.03443425	0.372	9959	tags=82%, list=60%, signal=205%
HALLMARK_MTORC1_SIGNALING	HALLMARK_MTORC1_SIGNALING	Details ...	150	0.20843798	0.20843798	0	0.03735411	0.42	6066	tags=57%, list=37%, signal=90%
HALLMARK_ANDROGEN_RESPONSE	HALLMARK_ANDROGEN_RESPONSE	Details ...	69	0.18844454	0.18844454	0.01333333	0.05777011	0.605	8162	tags=68%, list=49%, signal=134%
HALLMARK_ESTROGEN_RESPONSE_EARLY	HALLMARK_ESTROGEN_RESPONSE_EARLY	Details ...	128	0.1876152	0.1876152	0	0.05569345	0.611	3381	tags=39%, list=20%, signal=49%
HALLMARK_HEME_METABOLISM	HALLMARK_HEME_METABOLISM	Details ...	128	0.16871297	0.16871297	0	0.08437572	0.794	6405	tags=55%, list=39%, signal=90%
HALLMARK_PROTEIN_SECRETION	HALLMARK_PROTEIN_SECRETION	Details ...	67	0.16095105	0.16095105	0.03846154	0.09573174	0.859	8704	tags=69%, list=53%, signal=144%
HALLMARK_GLYCOLYSIS	HALLMARK_GLYCOLYSIS	Details ...	140	0.15935615	0.15935615	0.00208768	0.09476194	0.874	4947	tags=46%, list=30%, signal=65%
HALLMARK_DNA_REPAIR	HALLMARK_DNA_REPAIR	Details ...	109	0.14497328	0.14497328	0.01659751	0.12975691	0.953	10971	tags=81%, list=66%, signal=238%
HALLMARK_NOTCH_SIGNALING	HALLMARK_NOTCH_SIGNALING	Details ...	25	0.11290869	0.11290869	0.852459	0.28051132	1	11367	tags=80%, list=69%, signal=255%
HALLMARK_UV_RESPONSE_UP	HALLMARK_UV_RESPONSE_UP	Details ...	112	0.10488315	0.10488315	0.16393442	0.33316532	1	3888	tags=34%, list=24%, signal=44%
HALLMARK_WNT_BETA_CATENIN_SIGNALING	HALLMARK_WNT_BETA_CATENIN_SIGNALING	Details ...	34	0.08217726	0.08217726	0.9507874	0.5588368	1	1075	tags=15%, list=6%, signal=16%
HALLMARK_MYOGENESIS	HALLMARK_MYOGENESIS	Details ...	103	0.07996095	0.07996095	0.5294118	0.5641651	1	2860	tags=25%, list=17%, signal=30%
HALLMARK_UV_RESPONSE_DN	HALLMARK_UV_RESPONSE_DN	Details ...	103	0.06175308	0.06175308	0.76171875	0.78601754	1	5247	tags=38%, list=32%, signal=55%

Fig. S2 Hepatic lipid accumulation in EndoG KO mice.

(A) Liver histology of male EndoG WT and KO mice at 3 mo and 10 mo. Lipid accumulation is visualized by Oil Red O staining. (B) Liver triglyceride and serum chemistry of male EndoG WT and KO mice at 10 mo (n=10). (C) Whole body, liver weight and liver triglyceride levels in female EndoG WT and KO mice on HFD. (D) Gene Set Enrichment Analysis (GSEA) of EndoG WT and KO liver mRNA from mice fed HFD for 3 mo. *, $p<0.5$; **, $p<0.01$; ***, $p<0.001$.

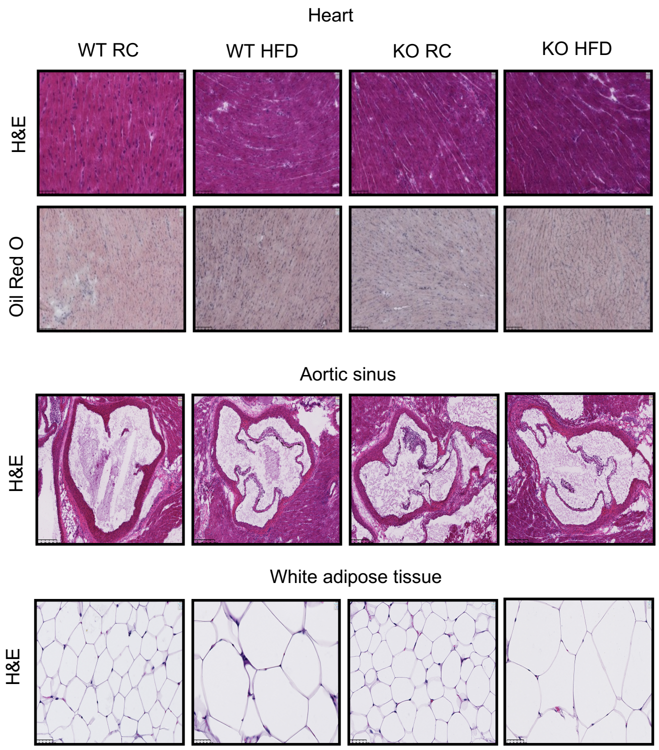

Fig. S3. Fat accumulation in tissues.

There is no difference in lipid accumulation in the heart, aortic sinus and white adipose tissue between male WT and EndoG KO mice on RC and HFD.

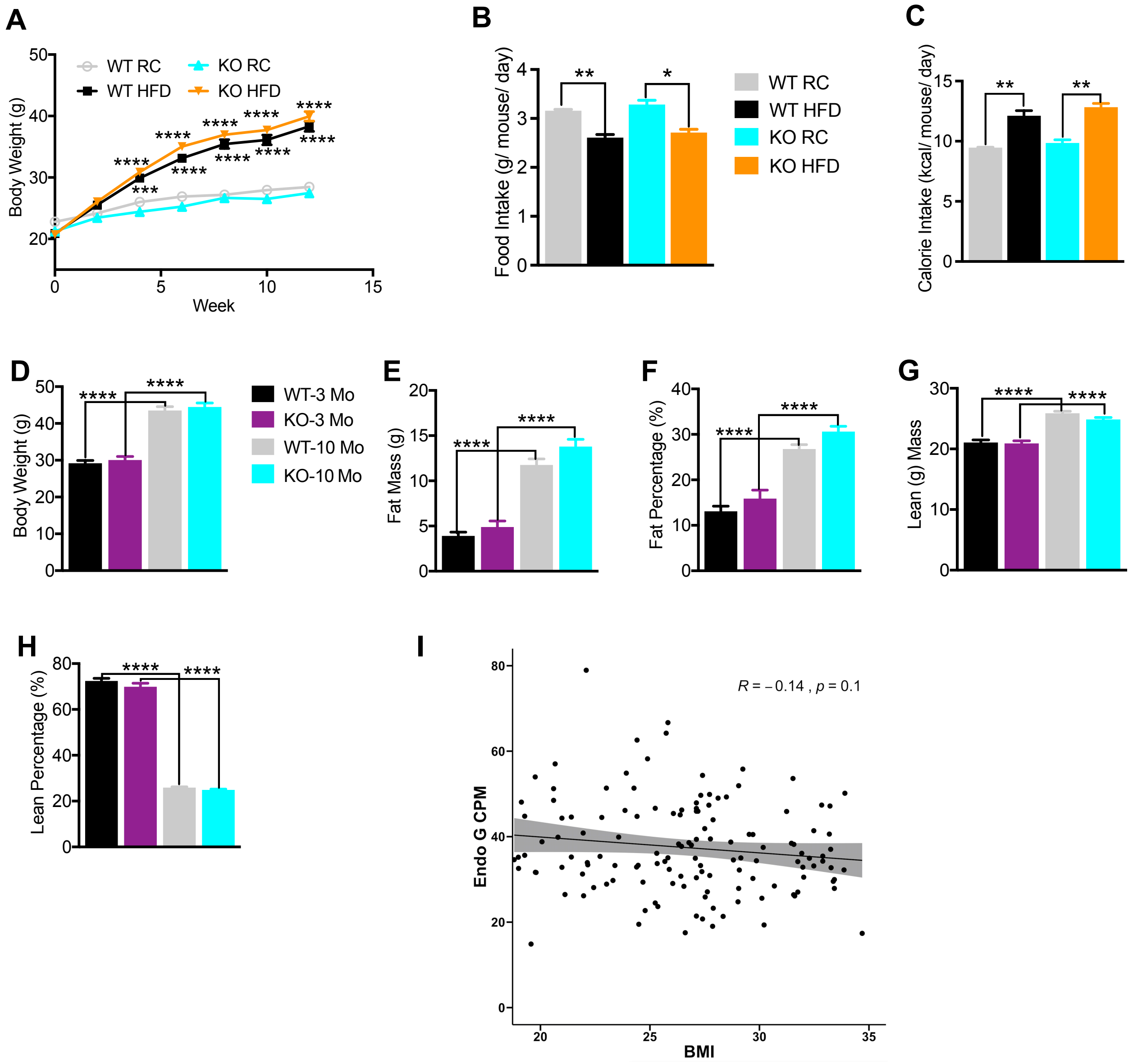

Fig. S4 EndoG levels and whole-body lipid accumulation, body composition or food intake. (A) Male mouse body weight at 3 mo on different diets (n=5-13), (B) food intake, (C) caloric intake, (D) body weight at 3 mo and 10 mo (n = 10 – 17 per group), (E) fat mass, (F) fat percentage, (G) lean mass, (H) lean mass percentage, (I) *EndoG* mRNA levels and BMI in the general population (men and women). *, *p*<0.5; **, *p*<0.01; ***, *p*<0.001; ****, *p*<0.0001.

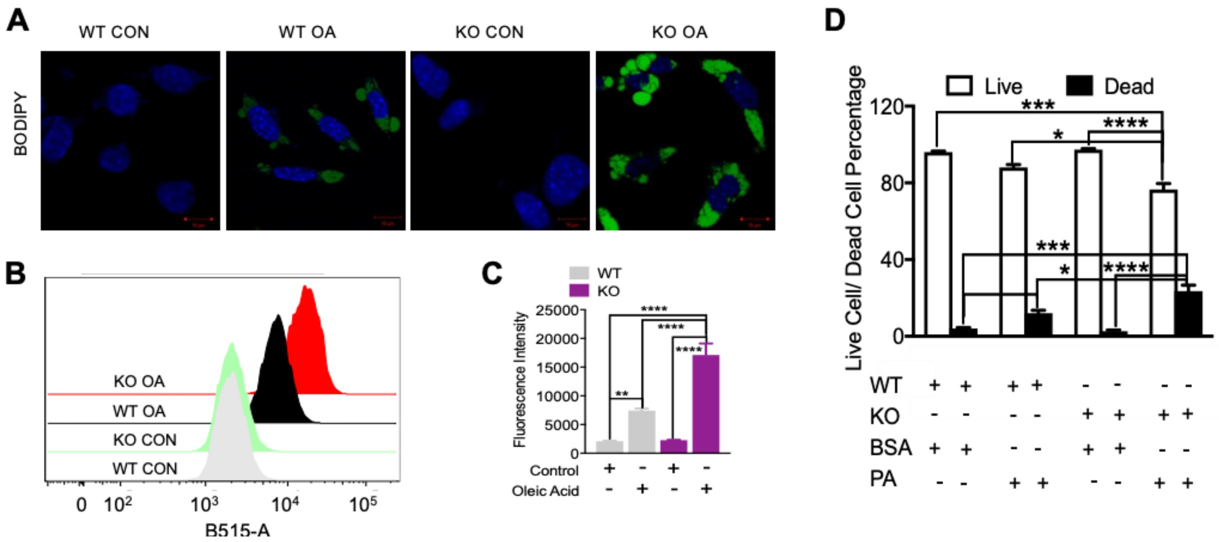

Fig. S5 EndoG promotes fat metabolism.

(A) Visualization of oleic acid (OA) accumulation in EndoG WT and KO MEFs by BODIPY staining followed by confocal fluorescence microscopy. Control (CON) is included for comparison. (B) BODIPY fluorescence of samples shown in (A) measured by flow cytometry. (C) Quantification of the flow cytometry result shown in (B). (D) Quantification of live and dead EndoG WT and KO MEF cells after they were treated with palmitic acid (PA, 0.3 mM) for 24 hrs (n = 3 - 6). The asterisks indicate significant differences by one-way ANOVA (*, p<0.05; **, p<0.01, ***, p<0.001, ****, p<0.0001).

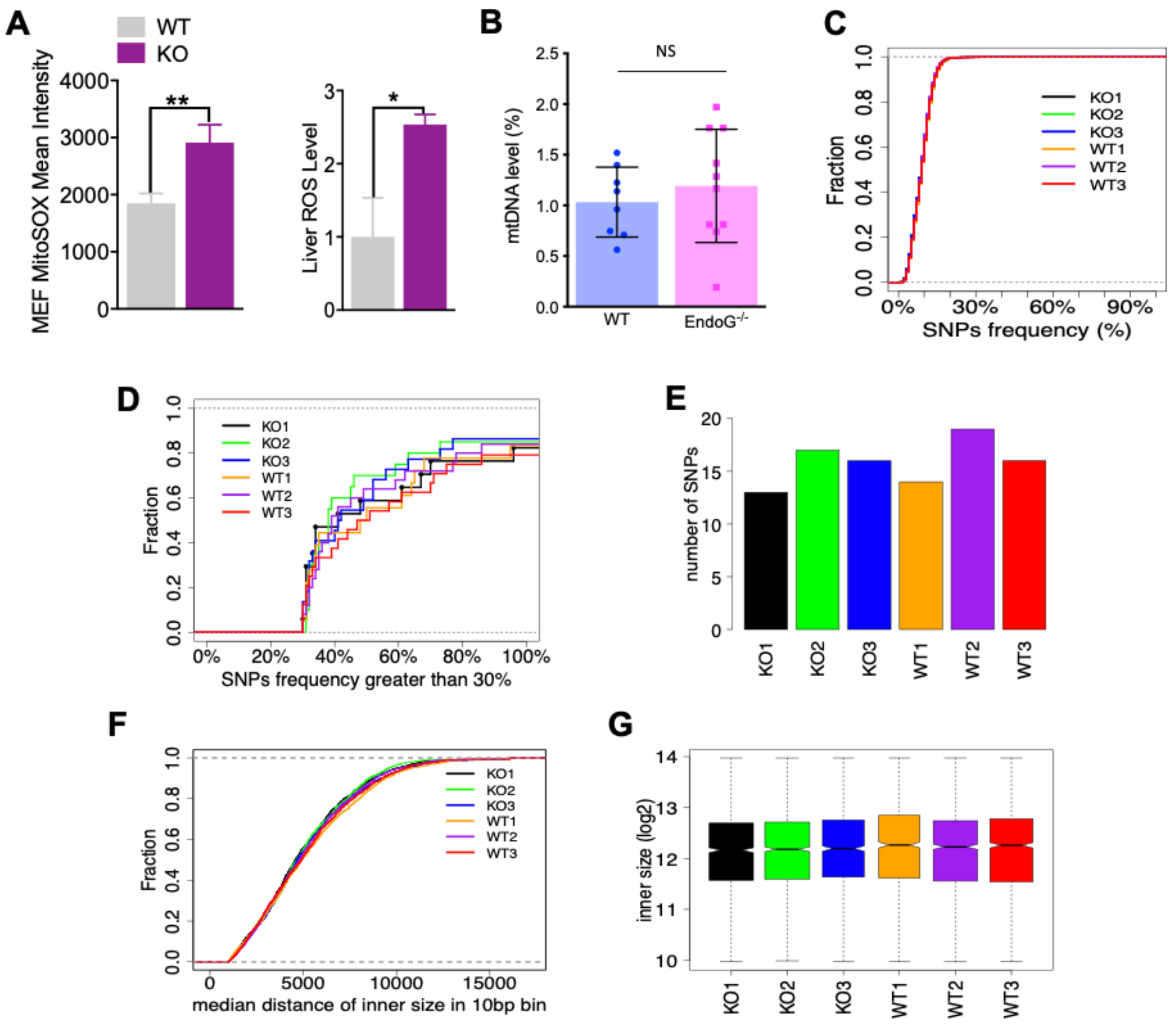

Fig. S6 EndoG is not required for mtDNA replication or mitochondrial genome stability.

(A) (left) Mitochondrial ROS was measured in EndoG WT and KO MEFs with MitoSOX red stain (5 μM) (n=5). (right) Liver ROS was measured with CM-H2DCFDA (25 μM) (n=5). (B) Mitochondrial DNA levels in EndoG WT and KO liver as measured by qPCR (n=5). (C) The empirical cumulative distribution plot of the SNPs mutation frequency between EndoG WT and KO triplicates samples. The plot shows the cumulative distribution of every single base mutation frequency on mitochondrial genome. There is no difference in SNPs mutation frequency distribution between the two conditions. (D) and (E) The subset of SNPs with the mutation frequency greater than 30%. (D) The empirical cumulative distribution of the subset of SNPs with the mutation frequency greater than 30%. (E) The bar graph of the number of SNPs with the

mutation frequency greater than 30% in each sample. The two plots show there is no significant difference between EndoG WT and KO mtDNA in the subset SNPs with higher mutation frequency. (F) The cumulative distribution of the inner sizes greater than 1 kb. The inner size is calculated based on the location of the two ends of each fragment. The inner sizes greater than 1 kb (the distance between the ends of R1 and R2 paired-end reads) were binned with 10 bp size windows. The plot shows the cumulative distribution of the median of each bin. (G) The boxplot shows the distribution of the inner size greater than 1 kb on log2 scale. There is no significantly different insertion or deletion between EndoG WT and knockout mtDNA. Data collected from three pairs of 12 months old EndoG WT and KO mice.

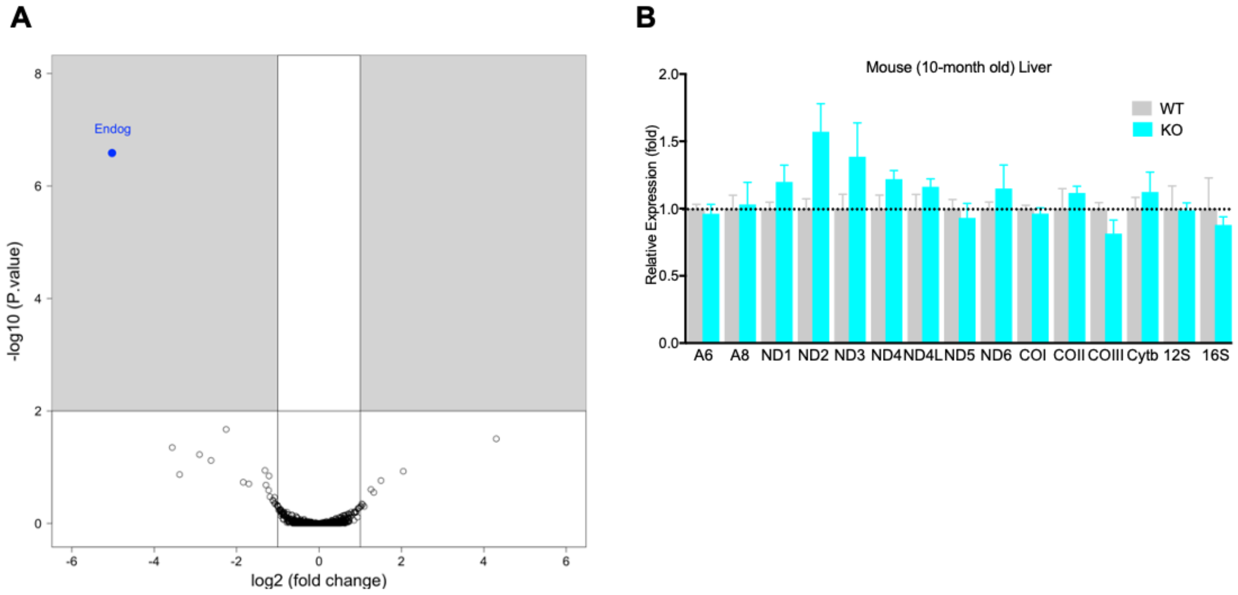

Fig. S7 EndoG does not affect nuclear or mitochondrial transcription in livers.

(A) Volcano plot of the transcriptomes of male EndoG WT and KO liver mRNA at 2-3 mo. The only significant difference between the two transcriptomes is the level of *endoG* mRNA. (B) Levels of mRNA and rRNA encoded by the mitochondrial genome in 10 mo EndoG WT and KO livers.

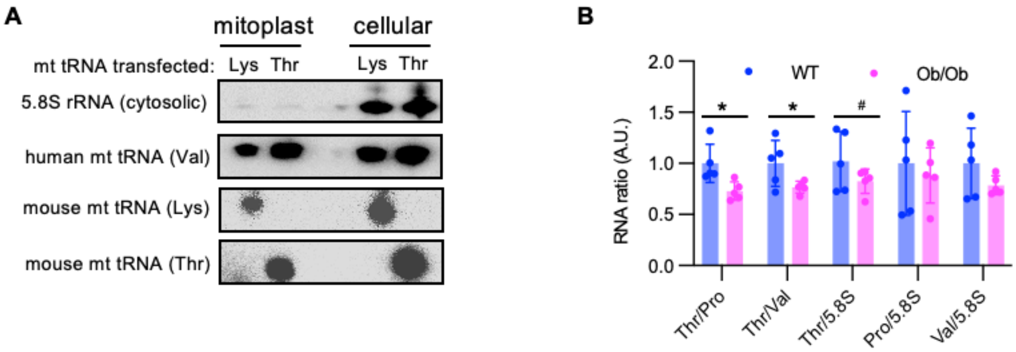

Fig. S8 EndoG function in mt tRNA^{Thr} production.

(A) Northern blot showing that transfected mouse mt-tRNA^{Lys} and mt-tRNA^{Thr} is delivered into the mitochondrial matrix (mitoplast) of human HeLa cells (see Fig. 3G-I). (B) Levels of mt-tRNAs in the liver of WT and *ob/ob* mice. *, p<0.05; #, p=0.06.

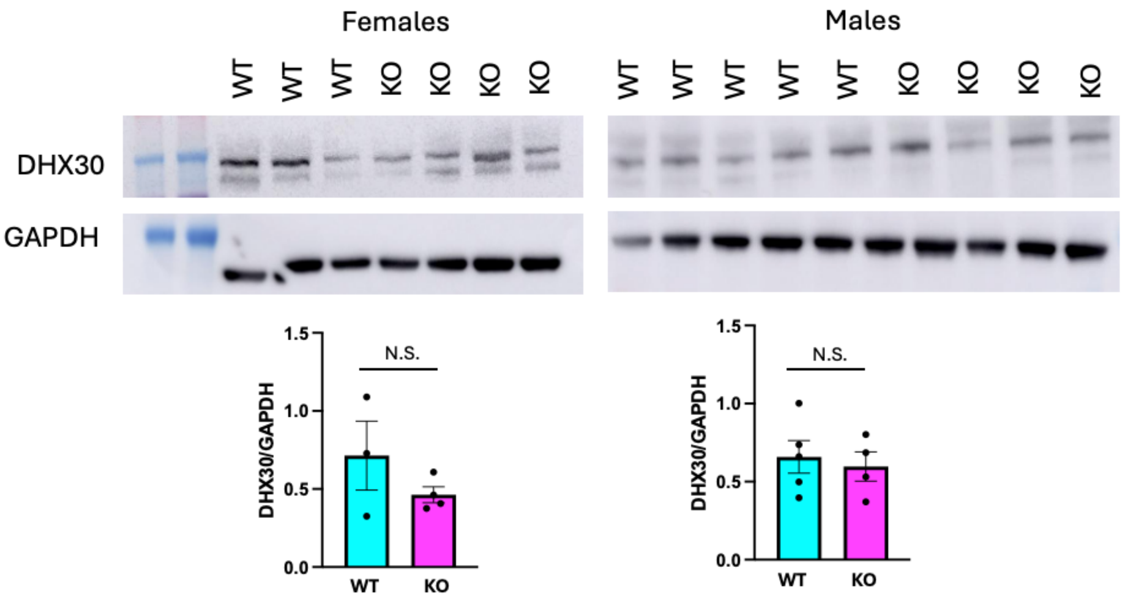

Fig. S9 DHX30 levels were not significantly affected by EndoG expression. Levels of DHX30 in the livers of female and male EndoG WT and EndoG KO mice on HFD are shown.
